# Phylodynamic reconstruction of major chicken infectious anemia virus clades epidemiology, dispersal, and evolution

**DOI:** 10.3389/fmicb.2025.1527335

**Published:** 2025-01-17

**Authors:** Giovanni Franzo, Matteo Legnardi, Francesca Poletto, Riccardo Baston, Giulia Faustini, Mattia Cecchinato, Claudia Maria Tucciarone

**Affiliations:** Department of Animal Medicine, Production and Health, University of Padua, Legnaro, Italy

**Keywords:** CIAV, molecular epidemiology, phylogenetics, phylodynamics, phylogeography, evolution, VP1 gene

## Abstract

**Introduction:**

Immunosuppressive diseases, such as chicken infectious anemia virus (CIAV), pose a major threat to livestock farming due to reduced disease resistance, poor vaccine response, and overall poor productivity. CIAV, recognized globally for decades, shows a significant genetic diversity, but its implications remain underexplored.

**Methods:**

This study analyzed over 1,000 VP1 sequences and examined CIAV’s epidemiology, evolution, and spread with various phylodynamic and phylogeographic approaches.

**Results:**

Findings suggest that CIAV likely originated in Japan in the early 20th century, followed by worldwide diversification in two main clades. Both clades exhibited no significant competition and similar global patterns, characterized by a progressive increase until about 2000, when a transient decline was observed for some years, potentially reflecting the increasing use of vaccines. Accordingly, although significant selective pressures were shaping viral evolution, comparable strengths were estimated in the two viral populations. The phylogeographic analysis identified several connections involving also distantly related regions, and more generally, multiple introduction events occurred in several countries and were followed by local evolution, indicative of unconstrained viral circulation.

**Discussion:**

Overall, the study highlights the ongoing circulation and evolution of different CIAV variants worldwide, where biosecurity measures and vaccination appear insufficient to prevent viral presence and dispersal.

## 1 Introduction

In both high- and low-income countries, poultry production is a cornerstone of agricultural economies, providing a cost-effective and culturally unrestricted protein source. In rural settings, chicken farming can significantly contribute to poverty alleviation through income generation and household food security (Mottet and Tempio, 2017; Windhorst, 2017). Poultry infectious diseases are thus a major hurdle not only for animals but also for human welfare and health. Among these, immunosuppressive diseases are of particular interest due to their ability to predispose to several secondary infections, requiring an intensification in antibiotic use, in addition to direct damages attributable to poor growth, increased mortality, carcass condemnations, etc. (Umar et al., 2014; Umar et al., 2017; Li et al., 2023).

Chicken infectious anemia virus (CIAV) is a member of the genus *Gyrovirus*, family *Anelloviridae*. It features a single-stranded, negative-sense circular genome of approximately 2.3 kb including 3 open reading frames (ORFs) that code for the proteins VP1, VP2, and VP3. VP1 is the main component of the viral capsid. It is involved in viral replication, virulence and is the target of neutralizing antibodies (Umar et al., 2014; Fatoba and Adeleke, 2019; Shah et al., 2023). The non-structural proteins VP2 and VP3 act as a scaffold to assist VP1 proper folding and as an inducer of apoptosis in chicken lymphoblastoid T and myeloid cells, respectively (Fatoba and Adeleke, 2019; Shah et al., 2023).

CIAV infection can lead to different outcomes depending on animal age and immune status. The virus affects hemocytoblasts and T-lymphocyte precursor cells, leading to immunosuppression. Subjects infected between 2–3 weeks of age, typically through vertical transmission, may show weakness, depression, anorexia, stunting, and ruffling. Increased mortality occurs in association with lymphoid atrophy, bone marrow aplasia, subcutaneous/intramuscular hemorrhages, and secondary infections (Di Francesco et al., 2021). Therefore, breeder vaccination, combined with adequate flock management and biosecurity, represent the main control strategy. When infection occurs at older ages, by oro-fecal or respiratory route, the disease is commonly subclinical, although immune depression may increase susceptibility to other infections and negatively affect the vaccination response (Schat, 2009).

CIAV was initially identified in Japan in 1979, soon after in Europe (1981), United States (1989) and South America (1991), and its worldwide distribution is now recognized (Techera et al., 2021; Shah et al., 2023). VP1 is commonly sequenced and used for strain characterization and molecular epidemiology studies because of its variability, largely due to a high mutation rate and recombination, to a lesser extent. Recently, Techera et al. (2021) reconstructed the origin and dispersal of CIAV based on 229 VP1 sequences, demonstrating an ancient origin and wide circulation of both clades. Examples of clade competition or differential evolution based on environmental/poultry productive systems in different areas have been reported in several studies dealing with other rapidly evolving viruses (Elena et al., 2001; Franzo et al., 2016, 2024a; Cheng et al., 2022). Although seminal, the work by Techera et al. was based on a relatively limited number of sequences. The present study aims to evaluate, based on a broadest VP1 dataset, the epidemiological history of CIAV, assessing and comparing the dynamics of the major clades, their potential competition, evolutionary patterns and differences.

## 2 Materials and methods

### 2.1 Sequence dataset preparation

Available CIAV VP1 coding sequences were downloaded from Genbank and aligned with MAFFT (Standley, 2013). Only sequences whose collection date and country were available and with adequate quality (i.e., absence of obvious misalignment, unknown bases, premature stop-codons or frameshift mutations) were maintained in the dataset (i.e., 1,532 sequences). Since the inclusion of complete VP1 only would lead to the exclusion of several sequences and relative collection regions and dates, a partial VP1 sequence dataset was generated aiming to include the highest number of sequences still retaining an informative genetic segment (i.e., 1,184 sequences). A preliminary tree was reconstructed using IQ-Tree (Nguyen et al., 2015) selecting the substitution model with the lowest Bayesian Information Criteria (BIC) calculated using the same software. All strains clustering together with vaccine ones, with high bootstrap support (i.e., >80), were identified and removed from the study. Recombination occurrence was assessed using GARD (setting the statistical significance level *p* < 0.05) (Kosakovsky Pond et al., 2006) and the strength of the phylogenetic signal was assessed through likelihood mapping analysis implemented in IQ-Tree (Nguyen et al., 2015), while the temporal signal was investigated using TempEST (Rambaut et al., 2016). Sequences deviating significantly from the expectations based on TempEST ‘root-to-tip’ regression analysis were also excluded being potentially misclassified vaccine or vaccine derived strains, or the result of significant sequencing errors, wrong record annotation, etc. A total of 1,151 sequences were thus retained in the study.

To perform the phylodynamic and phylogeographic analysis on CIAV, the partial VP1 dataset was down-sampled by randomly selecting a maximum of 5 strains for each country-year pair. This approach was useful to reduce the computational burden and enhance the following converging and mixing. At the same time, by performing repeated analysis on more balanced, but randomly generated datasets, it was possible to compensate and evaluate the effect of differential sequencing activity over time and space, as suggested by Layan et al. (2023).

Based on the divergence pattern observed through phylodynamic analysis, clade-specific datasets were also generated, and the same subsampling procedure was applied.

### 2.2 Phylodynamic and phylogeographic analysis

The generated datasets were analyzed to reconstruct several population parameters, including time to the most recent common ancestor (tMRCA), evolutionary rate, and viral population dynamics using the Bayesian serial coalescent approach implemented in BEAST 1.10.4 (Suchard et al., 2018). For each subset, the nucleotide substitution model was selected based on the BIC score calculated using JmodelTest (Darriba et al., 2012). The molecular clock was selected calculating the marginal likelihood estimation through path-sampling and stepping-stone methods, as suggested by Baele et al. (2012). The non-parametric Bayesian Skygrid was selected to reconstruct viral population changes over time (relative genetic diversity: Effective population size x generation time; N_e_x *τ*) (Hill and Baele, 2019). The CIAV spatial dispersal was reconstructed using the discrete state phylogeographic analysis as described by Lemey et al. (2009), selecting an asymmetric migration model combined with Bayesian stochastic search variable selection (BSSVS), to describe the most parsimonious spreading process and calculate a Bayesian Factor (BF) indicative of the statistical significance of the inferred migration path. Due to the high number of sampled locations, to obtain a more balanced dataset, countries were aggregated in macro-areas considering their spatial proximity and geopolitical factors (i.e., Africa, Asia, Central America, Europe, Middle East, North America, and South America). One run of 200 million generations was performed. Results were analyzed using Tracer 1.7 (Rambaut et al., 2018) and accepted only if the estimated sample size (ESS) was greater than 200 and the convergence and mixing were adequate after removal of the first 20% of data as burn-in. Parameter estimation was summarized in terms of mean and 95% highest posterior density (HPD). Maximum clade credibility (MCC) trees were constructed and annotated using TreeAnnotator (BEAST package). SpreaD4 (Bielejec et al., 2016) was used to calculate the BF associated with each migration route. All non-zero transition rates among countries were considered significant if the calculated BF was greater than 10. Additional summary statistics and graphical outputs were generated using homemade R scripts (Team RC, 2014). Comparable analysis were performed on clade specific datasets. However, a country level discrete state phylogeographic analysis was also performed in this case.

The average population size of the two clades over time was correlated using rolling window correlations between the two regular time series. The statistical significance of the rolling correlation coefficients was estimated using the NonParRolCor (Polanco-martínez and López-martínez, 2023) library of R, accounting for multiple testing effects via Monte Carlo simulations through a permutation approach. Different window lengths were evaluated in 2-year increments, and the correlation coefficient, critical value for the rolling correlation coefficients and significance [*p* < 0.05; i.e., the 95th quantile of the critical value] were assessed for each window.

### 2.3 Selective pressure analysis

The action of selective pressures was evaluated on the complete VP1 dataset, assessing the non-synonymous to synonymous substitution rate calculation (dN/dS). Pervasive selective pressures were analyzed using Fast Unconstrained Bayesian AppRoximation (FUBAR) (Murrell et al., 2013) and Fixed Effects Likelihood (FEL) (Kosakovsky Pond and Frost, 2005), while episodic selection occurrence was assessed with Mixed Effects Model of Evolution (MEME) (Murrell et al., 2012), implemented in HyPhy (Pond et al., 2005). The significance level was set at posterior probability (PP) > 0.9 and *p*-value <0.05 for FUBAR and FEL and MEME, respectively.

The difference in diversifying selective pressure strength among clades was evaluated using contrast-FEL (Kosakovsky Pond et al., 2021).

## 3 Results

### 3.1 Datasets

**Figure 1:**
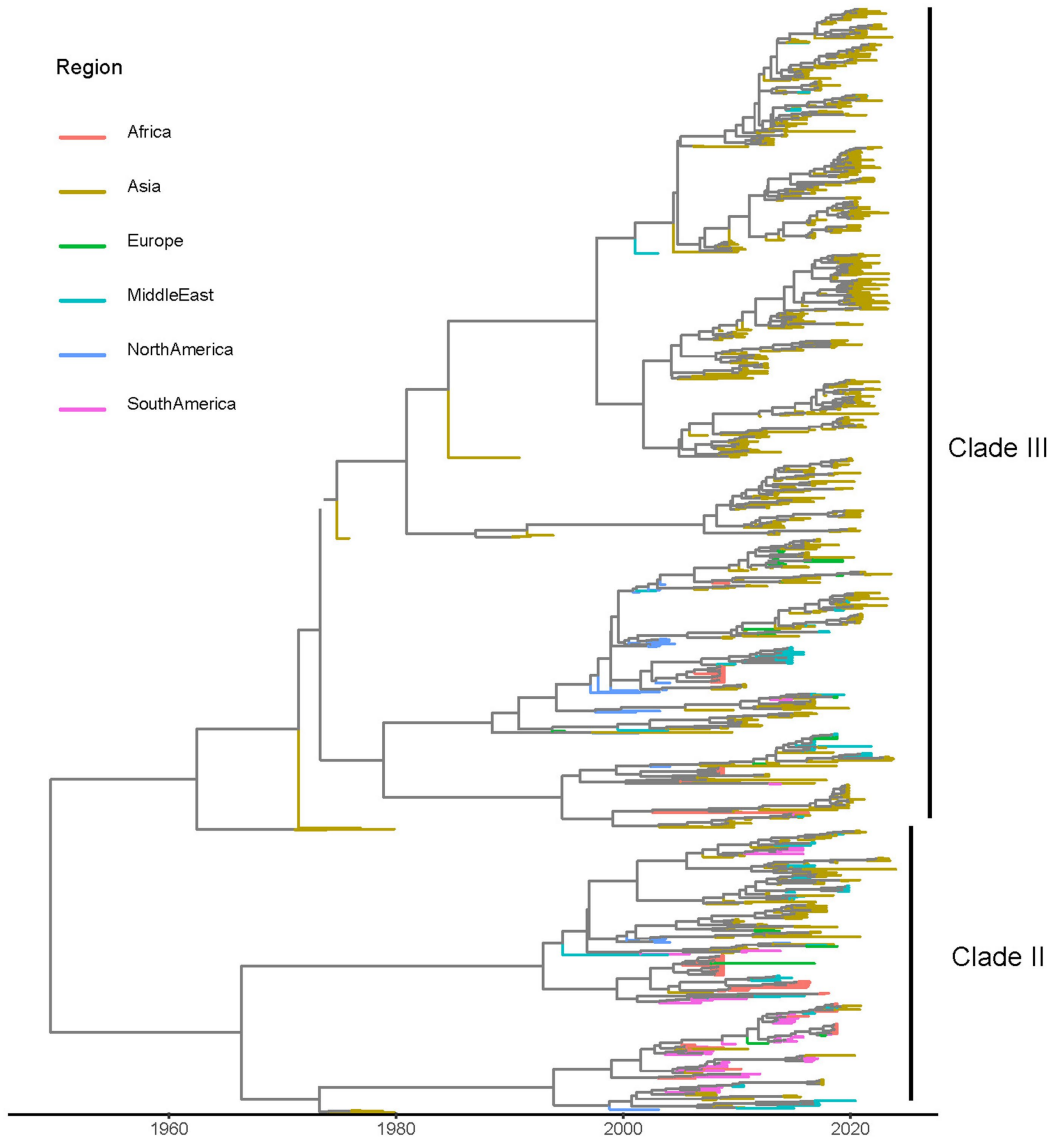
Maximum clade credibility (MCC) trees reconstructed based on the partial VP1 alignment. The terminal branches have been color-coded based on the region where samples were collected.

A total of 1,151 sequences were included in the final dataset; they originated from 31 countries, 6 macro-areas and were collected over a time interval from 1974 to 2024. Strains collected in different macro-areas were interspersed in the phylogenetic tree, although some sub-clades preferentially included strains from the same location (Figure 1). Because of the absence of clear clade definition criteria and the sometimes conflicting results of previous studies, only two clades, named Clade II and III, were considered for the present study purpose based on the results of MCC analysis (Figure 1), excluding the highly divergent Clade I, for which only two sequences distant more that 5% from other available strains were available. A summary of the included sequence features is reported in Supplementary Table S1.

### 3.2 Phylodynamic analysis

The tMRCA of all included strains, averaged among runs, was estimated to be 1945.715 [95HPD: 1895.07–1954.55], and the evolutionary rate was 5.72×10^−4^ [95HPD: 4.16×10^−4^-8.22×10^−3^].

Clade II specific tMRCA and evolutionary rate were 1972.44 [95HPD: 1962.70–1975.64] and 1.03×10^−3^ [95HPD: 7.17×10^−4^-1.43×10^−3^], respectively, while Clade III tMRCA was 1965.07 [95HPD: 1958.74–1969.74] and the evolutionary rate was 7.11×10^−4^ [95HPD: 5.69×10^−4^-8.74×10^−4^]. Minimal variability among runs was observed (Supplementary Figures S1, S2).

Similarly, the reconstruction of population dynamics revealed substantial overlap among randomly generated datasets. The CIAV viral population steadily increased from the tMRCA to about 2000, when a decrease occurred, followed by a rebound in 2010–2015.

**Figure 2:**
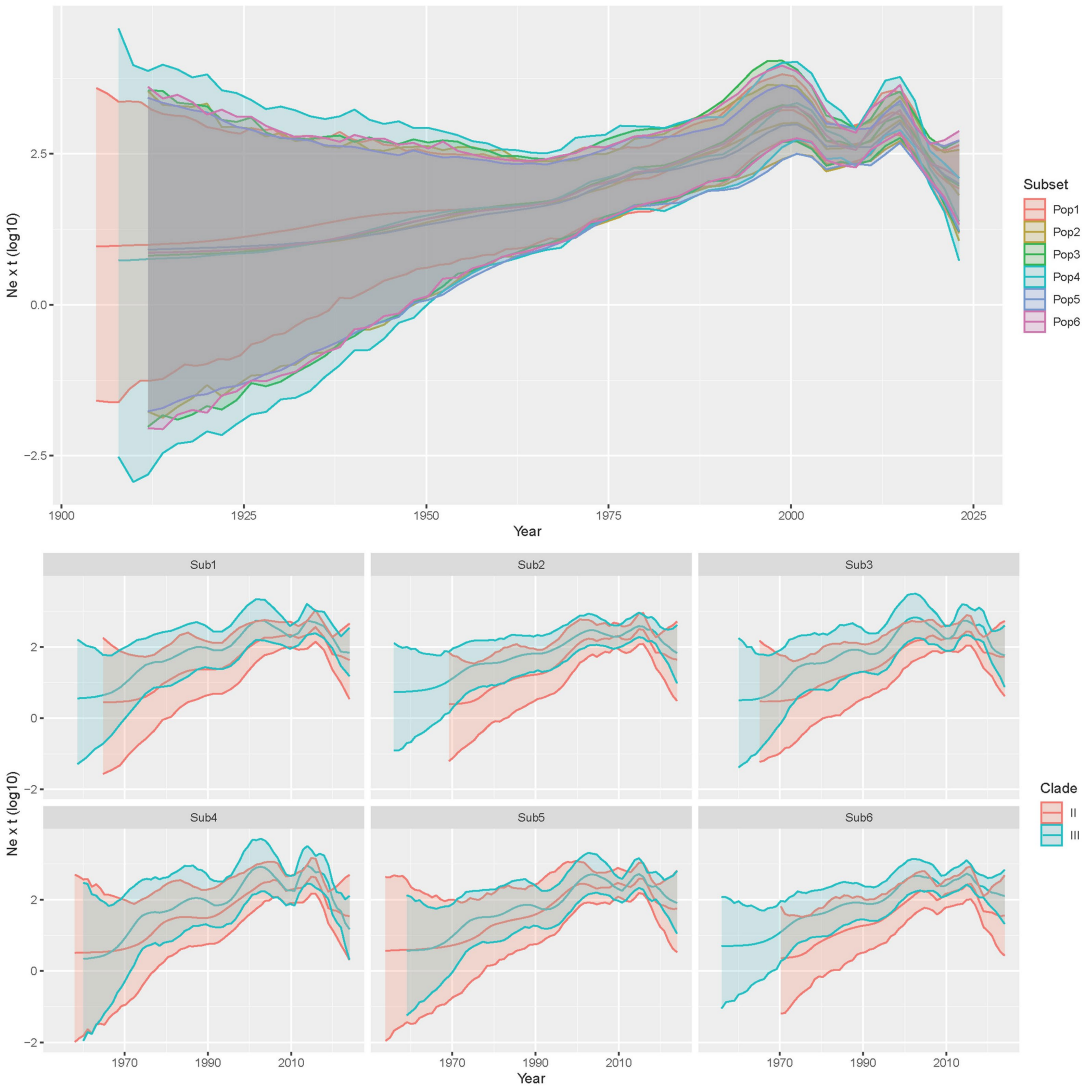
Upper figure: Mean relative genetic diversity (Ne x t) and relative 95HPD of the worldwide CIAV population over time. The results of the independent runs have been color-coded. Lower figure: Mean relative genetic diversity (Ne x t) and relative 95HPD of the CIAV clades over time population. The two clades have been color-coded while the results of independent, randomly generated datasets are provided in different panels.

An essentially comparable trend was observed for the two clades independently, although with minor variations among randomly generated datasets (Figure 2).

Clade III revealed an earlier rise and more apparent fluctuations over time, particularly evident around the 1980s-90s, 2000-05, and 2010-15. Clade II showed a steadier increase until about 2000, when the population tended to stabilize, even though fluctuations were still present. The rolling correlation highlighted an overall positive correlation between the population sizes of the two clades when considering both short and long time periods (Supplementary Figure S3).

### 3.3 Phylogeographic analysis

**Figure 3:**
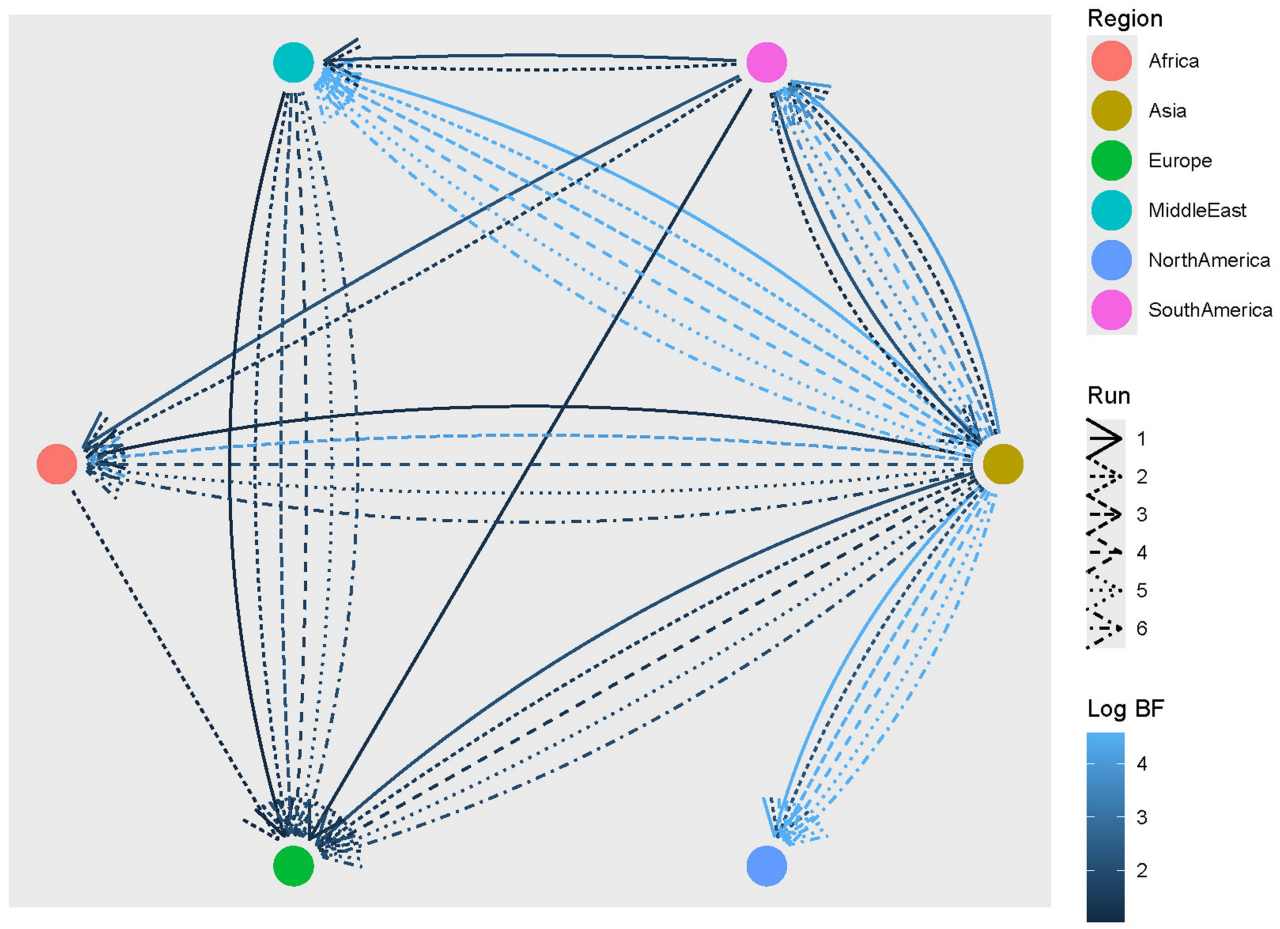
CIAV statistically supported migration rates. Edges among regions (color-coded) represent statistically supported migration rates. The color intensity is proportional to the BF value. The results of multiple runs are reported with different line styles.

The reconstruction of the CIAV migration process predicted a most likely origin in Asia, where the two clades diverged and thereafter spread to other continents from the 1960s. Overall, several connections among macro-areas were statistically well supported, with congruent scenarios among datasets (Figure 3; Supplementary Figure S4). Asia was the main source of viral dispersal, exporting strains to Africa, Europe, Middle East, North and South America. Europe was identified as the main importer, with connections to all locations except for North America. However, statistically supported migration rates were consistently inferred only from Asia and the Middle East.

The within-clade analysis confirmed a similar picture, with both groups originating in Asia, most likely in Japan, followed by worldwide dispersal.

However, especially for Clade II, overall low posterior probability characterized the estimation of ancestral locations, and relatively low among-dataset repeatability was observed. The initial stages of CIAV dispersal could thus hardly be reconstructed with confidence. Nonetheless, frequent transmission events were inferred, and although some country-specific clades were present, they were interspersed in the phylogenetic tree, testifying to multiple introduction events occurring over time (Supplementary Figures S5, S6).

The evaluation of clade-specific statistically significant migration rates confirmed the major role of Asia as a source of viral dispersal, although a reverse flow from North and South America was identified, particularly for Clade II. For this clade, South America was especially involved in the transmission of viral strains to other regions, including Africa and the Middle East. A bidirectional viral flow also occurred between Europe and these regions.

**Figure 4:**
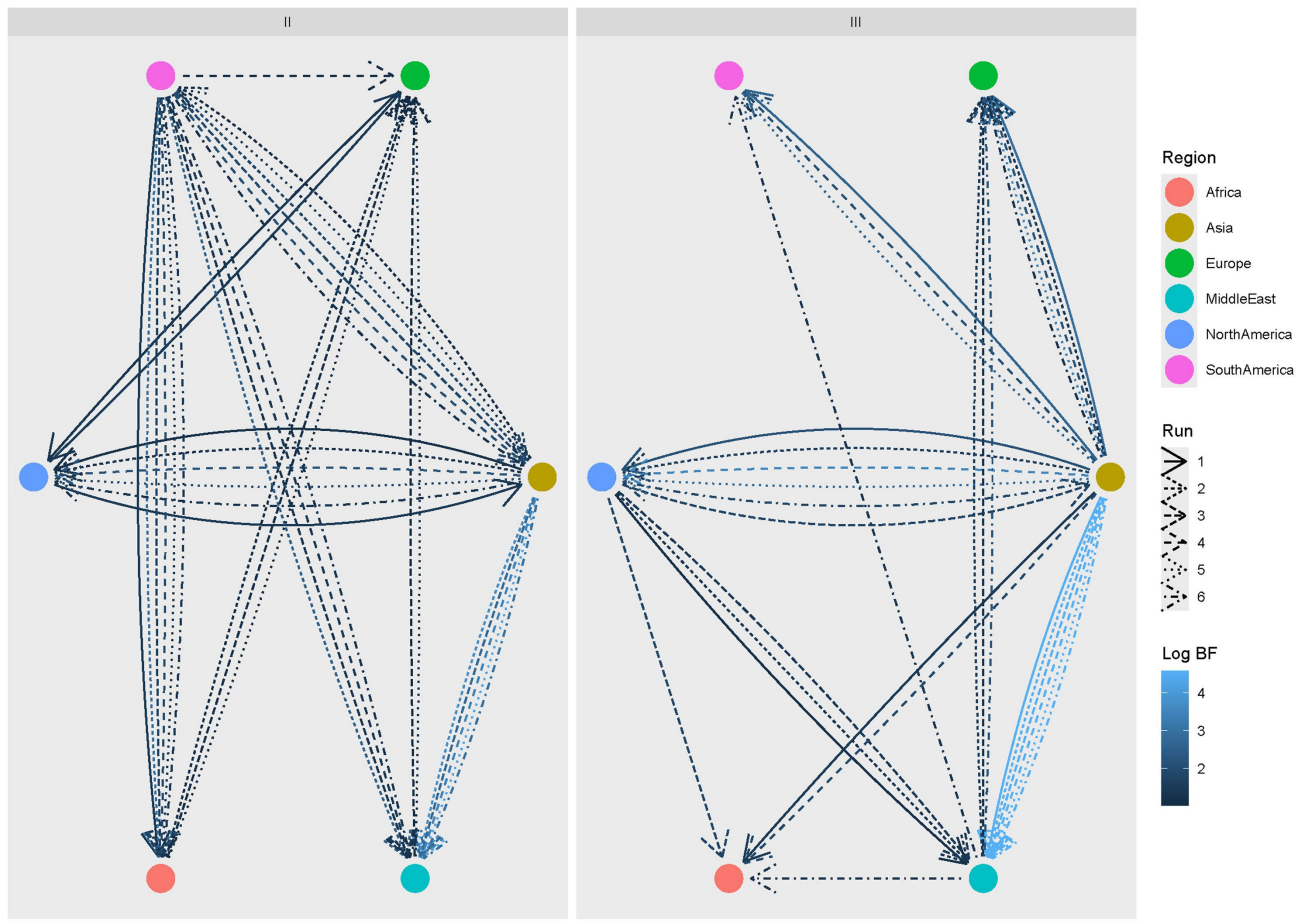
CIAV Clade II (left) and III (right) statistically supported migration rates. Edges among regions (color-coded) represent statistically supported migration rates. The color intensity is proportional to the BF value. The results of multiple runs are reported with different line styles.

Asia, on the other hand, was a major driver of Clade III spreading to other regions, both directly and indirectly, with the mediation of North America toward Africa and the Middle East. Similarly, the Middle East contributed to the introduction of Clade III in Europe (Figure 4).

### 3.4 Analysis of selective pressures

The analysis of selective pressures identified a limited number of sites under pervasive diversifying selection using FEL (i.e., 43, 157, 287, 290, 343, 370, and 413) and FUBAR (i.e., 22, 157, 287, 343, and 413). More codons were detected under episodic diversifying selection with MEME (i.e., 2, 3, 22, 26, 30, 31, 42, 105, 149, 157, 229, 242, 247, 269, 281, 287, 288, 343, 370, 376, and 413). However, when the strength of selection pressures was compared site-by-site between the two clades, a statistically significant difference was reported only at positions 21 and 141.

## 4 Discussion

CIAV is a significant pathogen for the poultry industry because of both direct and indirect damages of immunosuppression (Schat, 2009; Umar et al., 2014). Although reported almost simultaneously in Japan and Europe during 1979 and 1981, and eight years later in the United States (1989) (Techera et al., 2021), its control still represents a challenge due to its subtle and often subclinical course, which may hinder its diagnosis and favor its dispersal, as commonly occurs with other similar viruses of veterinary interest, like porcine circoviruses (Franzo et al., 2019, 2024c).

Techera et al. (2021) estimated an ancient origin at the beginning of the 20th century, followed by a progressive population expansion and dispersal. The present study builds on their valuable work and expands it, based on a dataset of over one thousand complete or nearly complete VP1 sequences, and aims at investigating CIAV epidemiological and evolutionary paths. The present work does not aim to establish sub-species classification criteria, but to reconstruct the history, epidemiological and evolutive trends of CIAV groups of primary relevance. Therefore Clades II and III were defined and selected based on the branching pattern of MCC trees. Notably, the two clades, as presently defined, diverged approximately in the same period, thus allowing for the investigation and comparison within a common timeframe. The highly divergent Clade I was not included due to the negligible number of strains, which prevented any dedicated analysis and suggested the limited relevance of this viral group. The previously defined Clade IV was not considered as an independent group because of the inconsistency in its topological relationship with other clades already reported in previous studies (Ou et al., 2018; Di Francesco et al., 2021; Shah et al., 2023; Song et al., 2024). Thus, Clade IV was considered an atypical variant rather than a different clade as proposed also by other authors (Techera et al., 2021). Sequences available from GenBank originated from several other studies, performed with different purposes. Therefore, the effect of sampling bias represents one of the main limitations of the present work (Hall et al., 2016). By generating several random sequence datasets, it was possible to demonstrate that, despite the unavoidable limitations intrinsically linked to the sparse data available, consistent results could still be obtained.

The origin of CIAV was estimated in Asia in the 1960s, with Japan having the highest posterior probability, in agreement with previous studies (Techera et al., 2021). Thus, after its origin, the virus likely circulated locally for decades, progressively diverging before spreading intercontinentally. The substitution rate was also confirmed to be exceptionally high, in the range of rapidly evolving ssDNA viruses like PCV2 (Franzo et al., 2016).

As for other viruses, Asia was estimated to be the main source of dispersal, both directly (Techera et al., 2021) and through the mediation of other countries. The Middle East was confirmed as a significant step for pathogen introduction into Europe, as previously reported for other avian viruses (Houta et al., 2021; Franzo et al., 2024b). The geographical location bridging Asia and Europe, along with the cultural and economic relationships with both regions, can easily explain this finding. Similar intricate networks were inferred at both macro-area and country levels for the two clades, testifying to largely unconstrained viral circulation (Shah et al., 2023), and such a pattern is particularly commonly described for diseases often having subclinical course (Klaumann et al., 2018; Franzo et al., 2020). Regardless of the clade, strains sampled in the same country often formed several independent clades interspersed in the phylogenetic tree, highlighting the occurrence of multiple introduction events at different time points, followed by local independent evolution (Ou et al., 2018; Shah et al., 2023). Although the overall migration patterns and trends can be considered accurate, as supported by their repeatability across datasets, the incomplete sampling (i.e., the likely absence of sequences from several countries and time periods due to limited diagnostic and sequencing activity) warrants caution when interpreting specific region-to-region or country-to-country contacts, as several hidden links may have been missed.

Taken together, these pieces of evidence suggest the inefficacy of measures in preventing viral dispersal, even over long distances, and in limiting and contrasting local persistence (Shah et al., 2023). While spreading, the two clades showed a progressive population increase until about 2000, which likely mirrors the progressive expansion of the poultry sector combined with the viral geographical spread, invading new host populations. Thereafter, both clades showed a contraction at the end of the 1990s, lasting about 10 years and more marked for Clade III. The progressive introduction and use of CIAV vaccines during the 1990s might explain the reduction of the viral population. This flection in CIAV population was not observed by Techera et al. (2021) that reconstructed a steady increase, although a certain stabilization of the effective population size in the considered period, followed by a sharper increase, was observed in that study also. The lower number of available sequences in Techera et al. (2021) could have decreased the resolution in the skyline estimation. Clade III includes all vaccine strains developed to date, and a selectively higher cross-protection could be speculated, leading to the more evident decline of this clade. Unfortunately, this field has been poorly investigated experimentally and any conclusion would be speculative. However, a population size rebound was observed for both clades after 2010, and again in this case, the phenomenon was more evident for Clade III. Whether this is due to episodes of vaccine escape or to the further expansion of poultry farming and relative international connections overcompensating the vaccine application remains to be established. CIAV circulation in vaccinated animal populations has been widely demonstrated, strengthening the latter hypothesis (Quaglia et al., 2021). Moreover, several field evidences suggest that vaccine strains could revert to virulence and vaccine-derived strains might circulate in the field (Erfan et al., 2018; Ou et al., 2018; Quaglia et al., 2021). Even though strains closely related to vaccines were removed from the dataset, a precise distinction can be challenging, especially if a long circulation and evolution in the field occurred. The persistence of these misclassified strains might also have inflated our estimations.

The analysis of selective pressures detected several sites under diversifying selection, especially episodic selection. The lack of an experimentally determined structural model of the VP1 prevents a precise spatial analysis of the amino acids subjected to selective constraints. Nevertheless, considering the VP1 role in capsid composition, a role of immune-driven selection can be hypothesized (Fatoba and Adeleke, 2019).

When the strength of selective forces was compared codon-by-codon between the two clades, only two sites were proven to be under significantly different selection. Based on these findings, the role of vaccination as a major driver of virus evolution appears weakened. Moreover, differently from what has been observed for other viruses characterized by multiple clades sharing the same environment (Franzo et al., 2016, 2023), no apparent competition between Clades II and III emerged from the present study. On the contrary, the two clades shared common dispersal patterns, highly correlated population dynamics over time, and evolutionary pressures. Comparable biological and pathogenic features, as well as substantial cross-reactivity, can thus be inferred.

## 5 Conclusion

Overall, the present study describes the wide and increasing presence of different CIAV variants over time in different regions of the world. Interestingly and unlike other pathogens, no competition between the considered clades was detected, suggesting overlapping immunological features, also supported by the similar selective forces acting on the two groups and comparable response to vaccination introduction. The remarkable viral spreading indicates the limited efficacy of both biosecurity and vaccination, at least in preventing viral presence and dispersal.

## Data Availability

The original contributions presented in the study are included in the article/Supplementary material, further inquiries can be directed to the corresponding author.
