# Supplementary material for: Phylodynamic reconstruction of major chicken infectious anemia virus clades epidemiology, dispersal, and evolution
Source: Front Microbiol. 2025 Jan 17;16:1527335. doi: 10.3389/fmicb.2025.1527335 (PMC11782247; doi:10.3389/fmicb.2025.1527335)
Supplement: Supplementary file 2 [file Supplementary_file_1.docx]

Supplementary Material


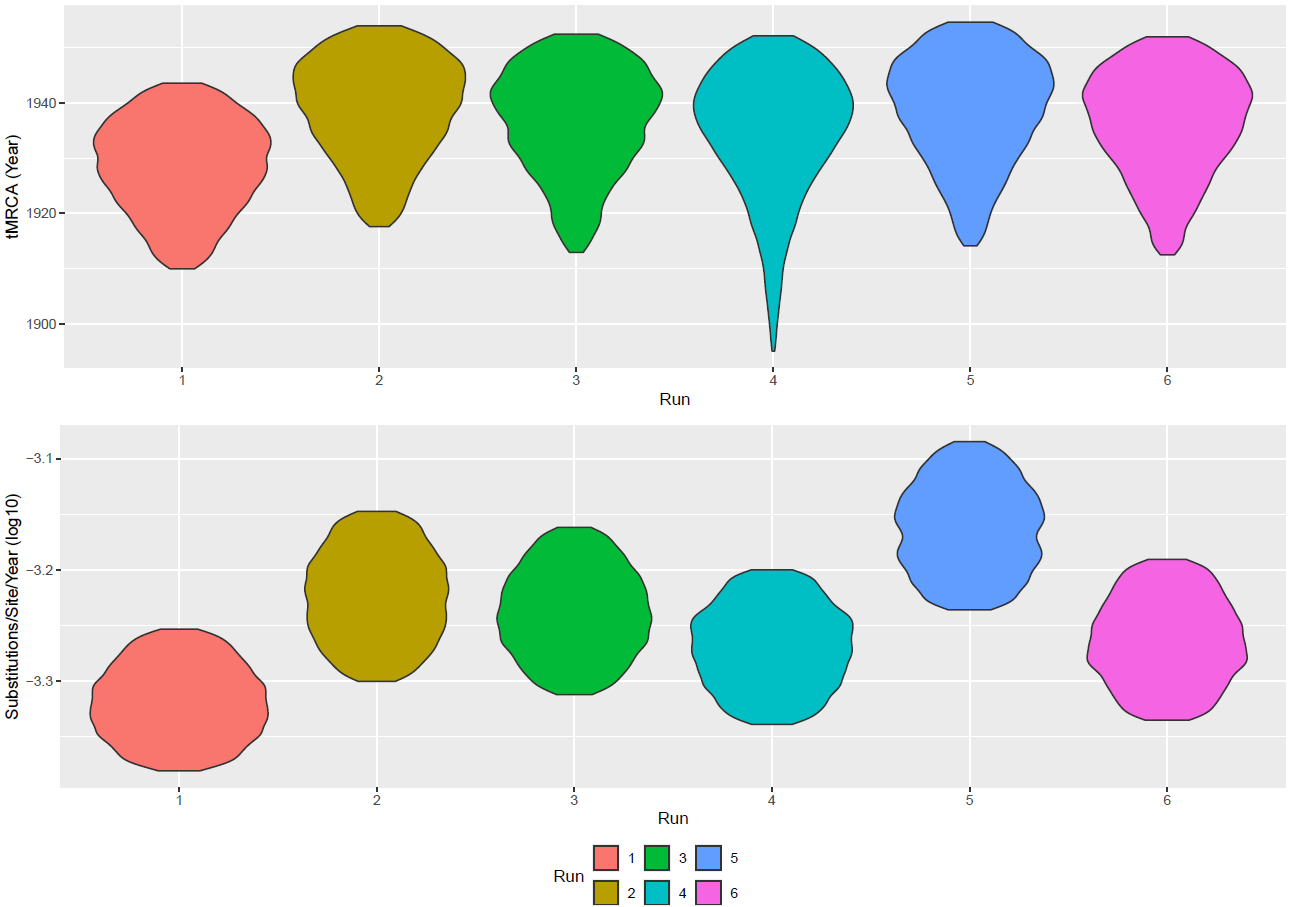


**Supplementary Figure 1.** Violin plot reporting the estimated 95HPD of tMRCA (upper panel) and evolutionary rates (lower panel) of CIAV. The results of the 6 randomly generated datasets have been depicted with different colours.


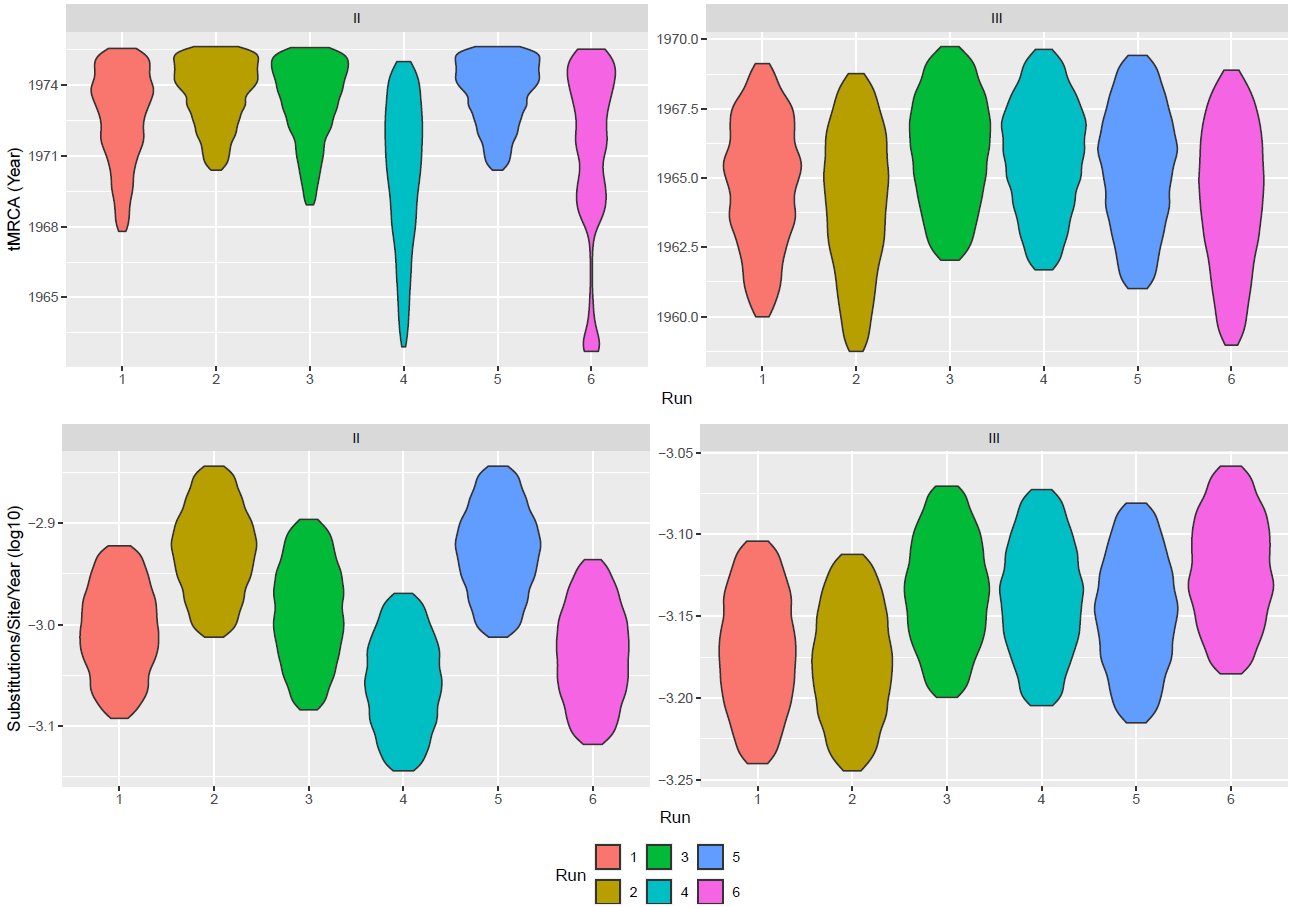


**Supplementary figure 2.** Violin plot reporting the estimated 95HPD of tMRCA (upper panel) and evolutionary rates (lower panel) of the Clade II (left panels) and III (right panels) of CIAV. The results of the 6 randomly generated datasets have been depicted with different colours.


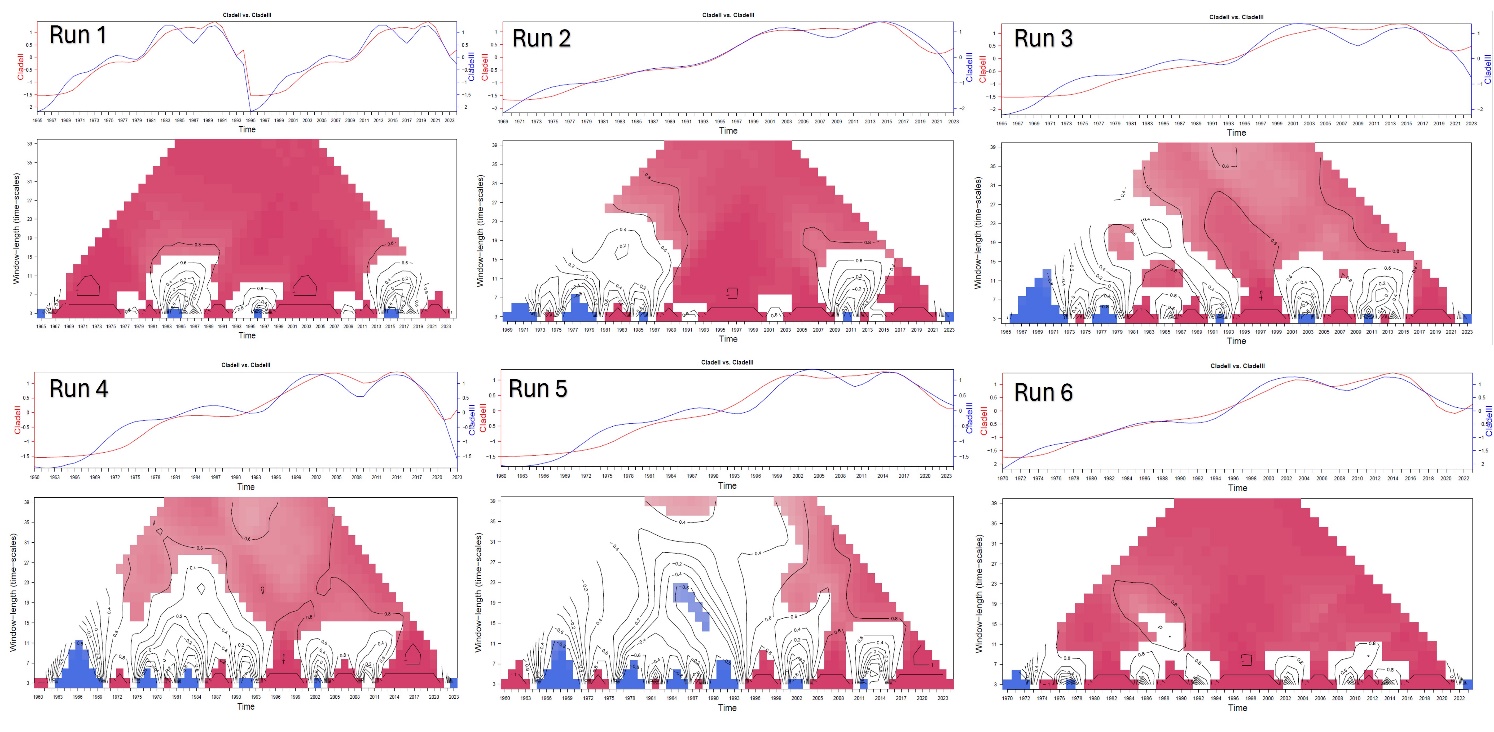


**Supplementary figure 3.** Results of the rolling correlation coefficient analysis among viral population size of Clade II and III. For each dataset, reported in different panels, the upper panel reports the trend (centered and scaled) of both variables while in the bottom one, the heatmap reports the rolling correlation coefficients calculated for different years and window sizes. The strength of the correlation has been color-coded while not statistically significant (95% confidence level) coefficients are left blank. Line contours indicate similar values of rolling correlation coefficients.


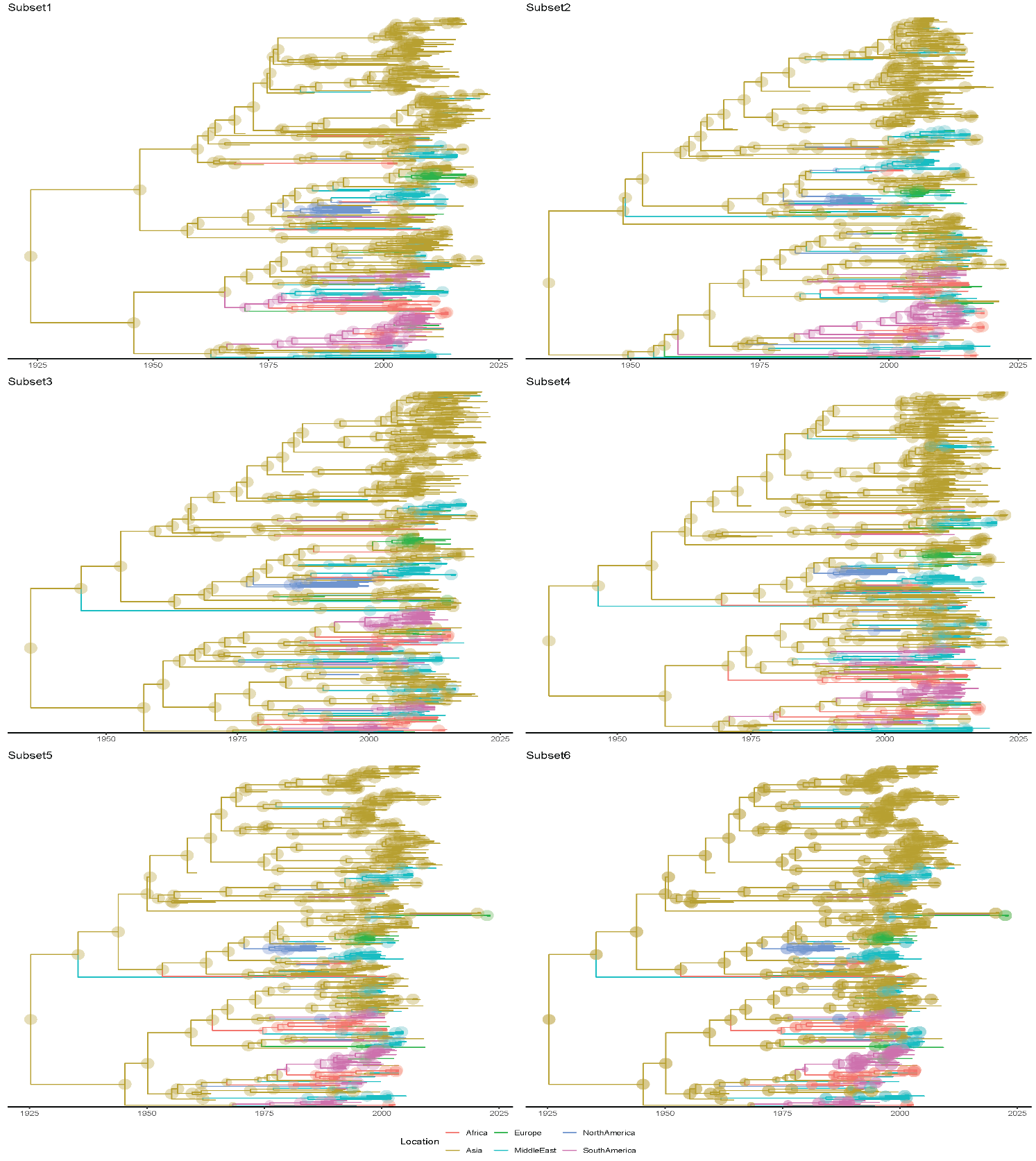


**Supplementary figure 4.** Maximum clade credibility trees based on the CIAV partial VP1 dataset. Tips and branches are color-coded according to the collection region or the one estimated with the higher posterior probability, respectively. Node size is proportional to the posterior probability of the inferred locations. The results of different datasets are reported in different panels. Higher-resolution images are provided in the supplementary figure .zip file.


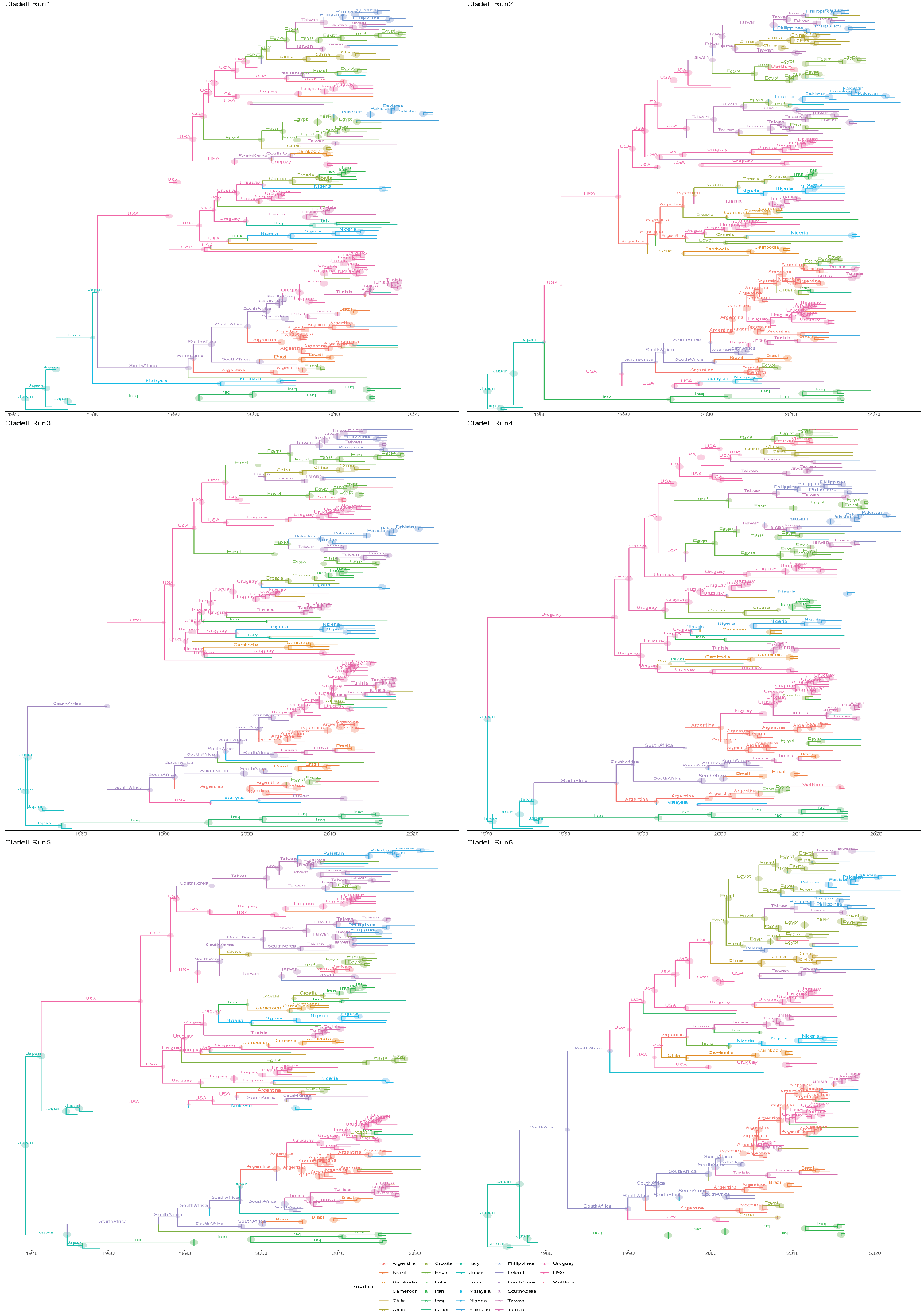


**Supplementary figure 5**. Maximum clade credibility trees based on the CIAV Clade II partial VP1 dataset. Tips and branches are color-coded and named according to the collection country or the one estimated with the higher posterior probability, respectively. Node size is proportional to the posterior probability of the inferred locations. The results of different datasets are reported in different panels. Higher-resolution images are provided in the supplementary figure .zip file.


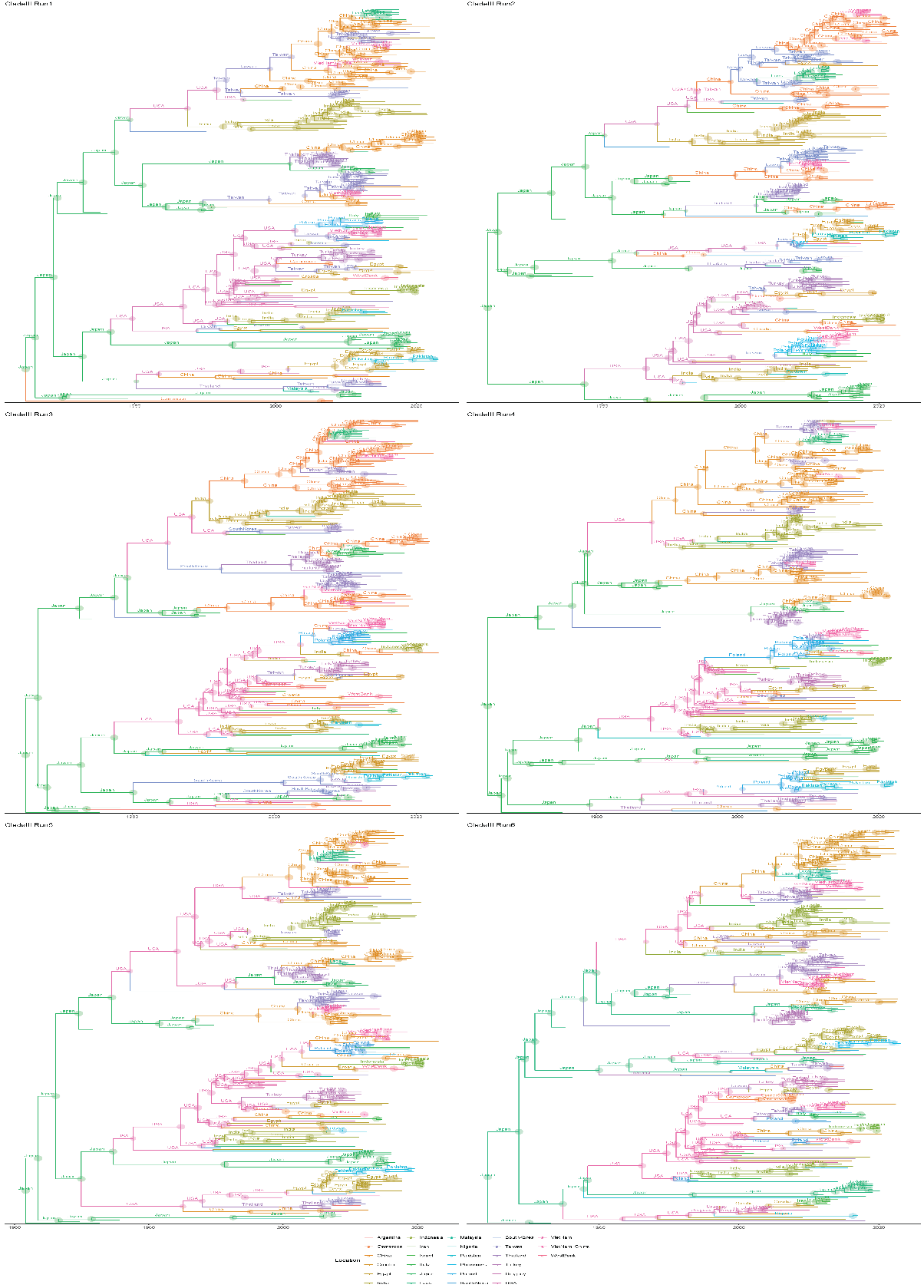


**Supplementary figure 6.** Maximum clade credibility trees based on the CIAV Clade III partial VP1 dataset. Tips and branches are color-coded and named according to the collection country or the one estimated with the higher posterior probability, respectively. Node size is proportional to the posterior probability of the inferred locations. The results of different datasets are reported in different panels. Higher-resolution images are provided in the supplementary figure .zip file.
