# Supplementary material for: Phylodynamic reconstruction of major chicken infectious anemia virus clades epidemiology, dispersal, and evolution
Source: Front Microbiol. 2025 Jan 17;16:1527335. doi: 10.3389/fmicb.2025.1527335 (PMC11782247; doi:10.3389/fmicb.2025.1527335)
Supplement: Supplementary file 3 [file Data_Sheet_1.zip › Supplementary figure 6.pdf]

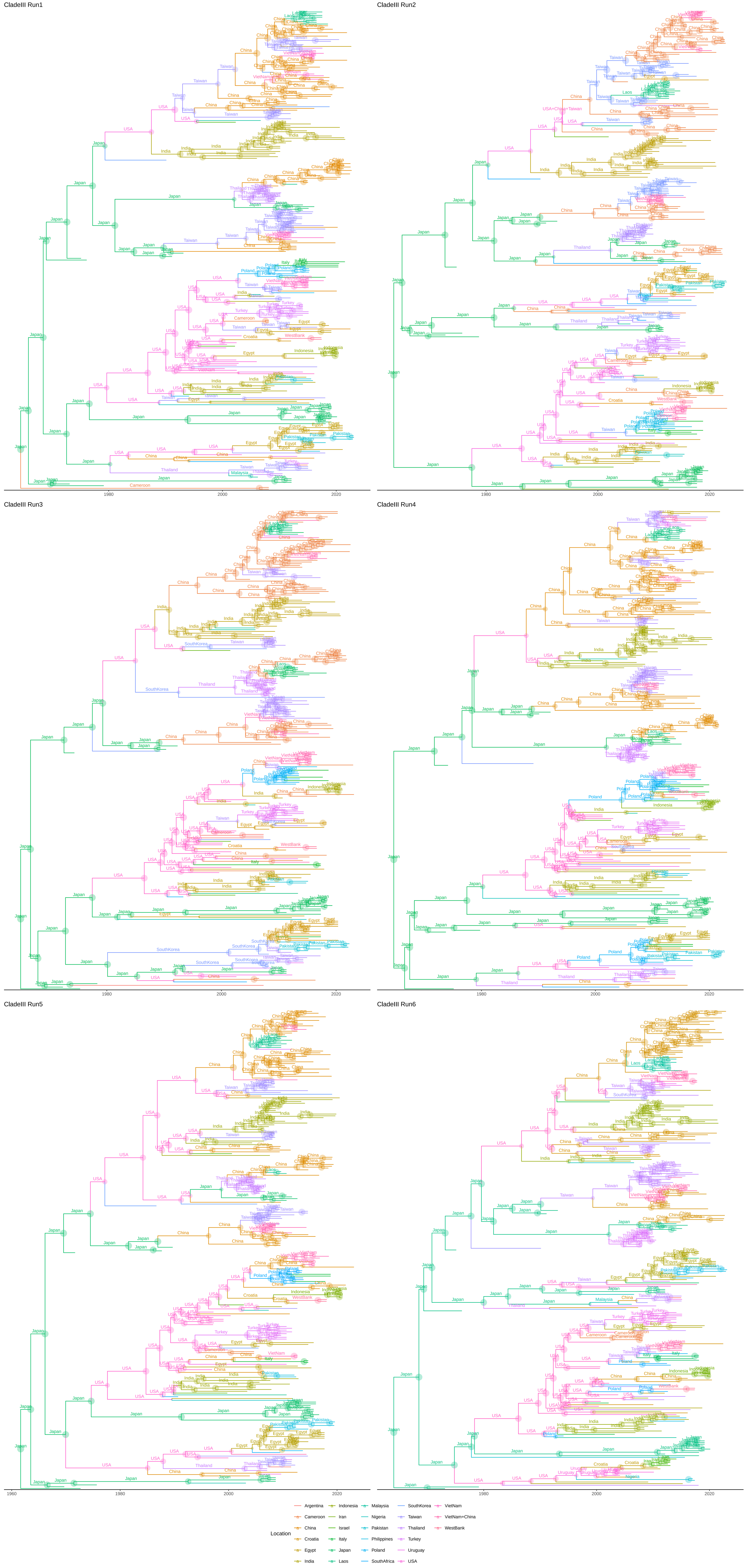
CladeIII Run1
CladeIII Run2
CladeIII Run3
CladeIII Run4
CladeIII Run5
CladeIII Run6
1960
1980
2000
2020
Location
Argentina
Cameroon
China
Croatia
Egypt
India
Indonesia
Iran
Israel
Italy
Japan
Laos
Malaysia
Nigeria
Pakistan
Philippines
Poland
SouthAfrica
SouthKorea
Taiwan
Thailand
Turkey
Uruguay
USA
VietNam
VietNam+China
WestBank
