# Supplementary figures and images for: Phylodynamic reconstruction of major chicken infectious anemia virus clades epidemiology, dispersal, and evolution
Source: Front Microbiol. 2025 Jan 17;16:1527335. doi: 10.3389/fmicb.2025.1527335 (PMC11782247; doi:10.3389/fmicb.2025.1527335)

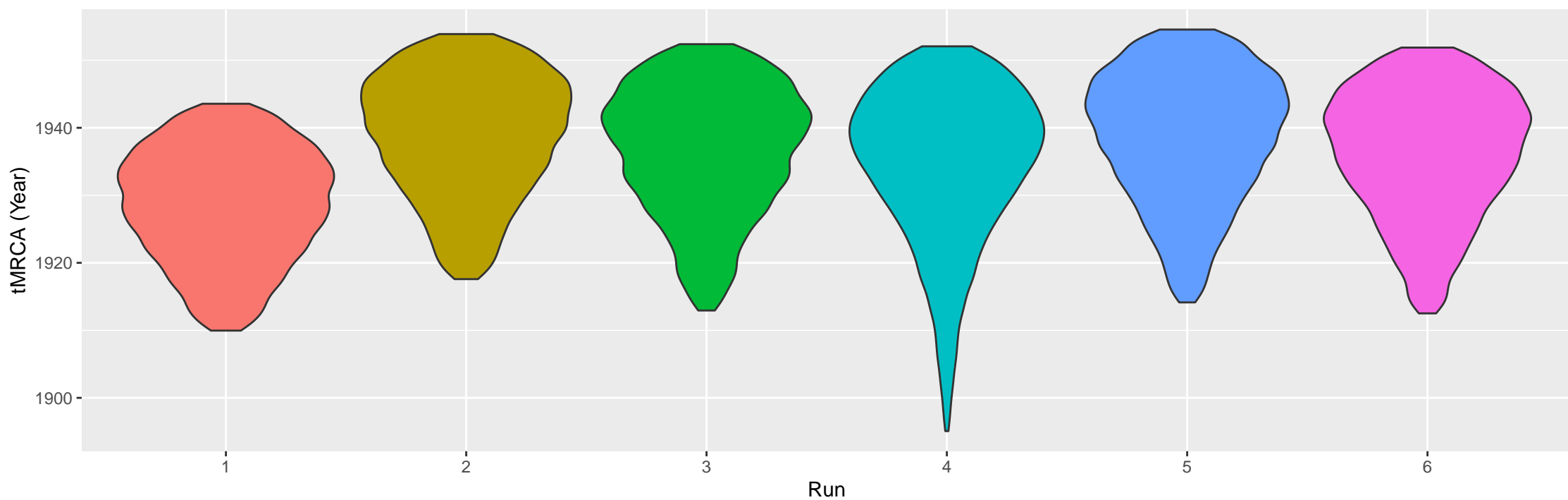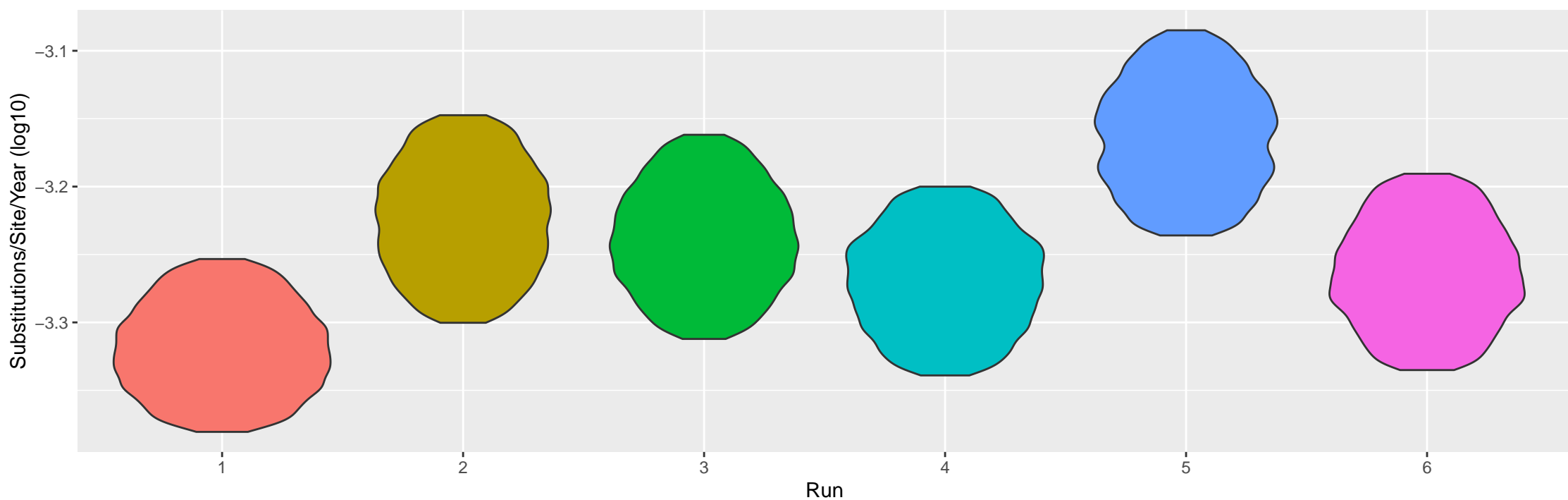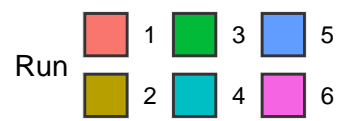

Supplement: Supplementary file 3 [file Data_Sheet_1.zip › Supplementary figure 1.pdf]

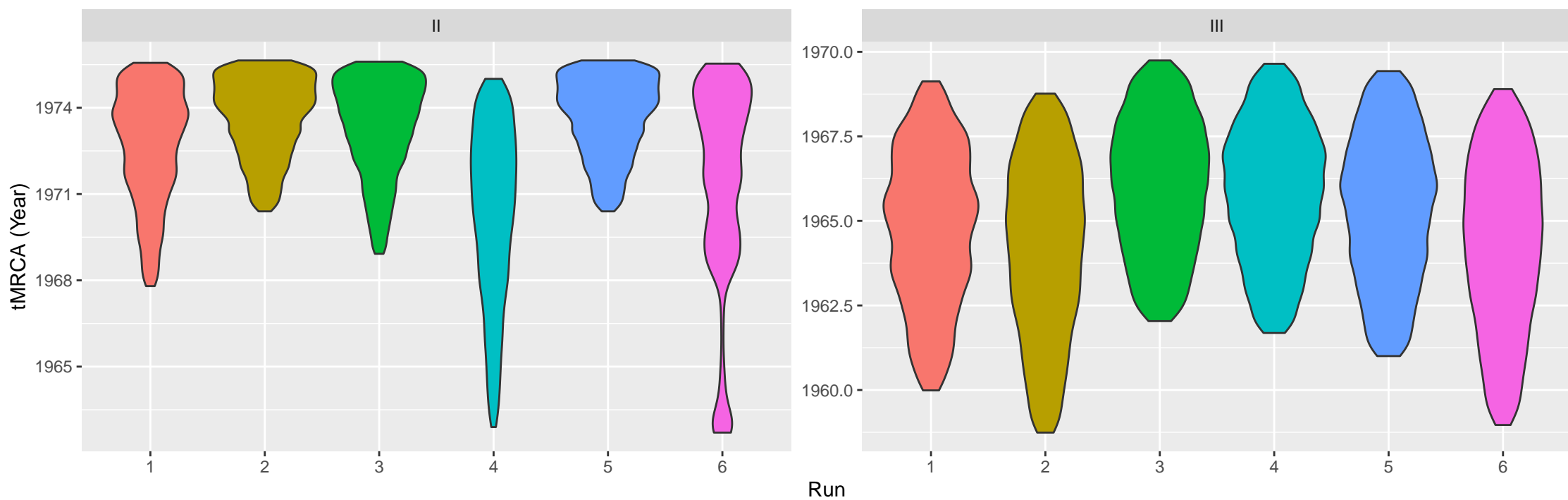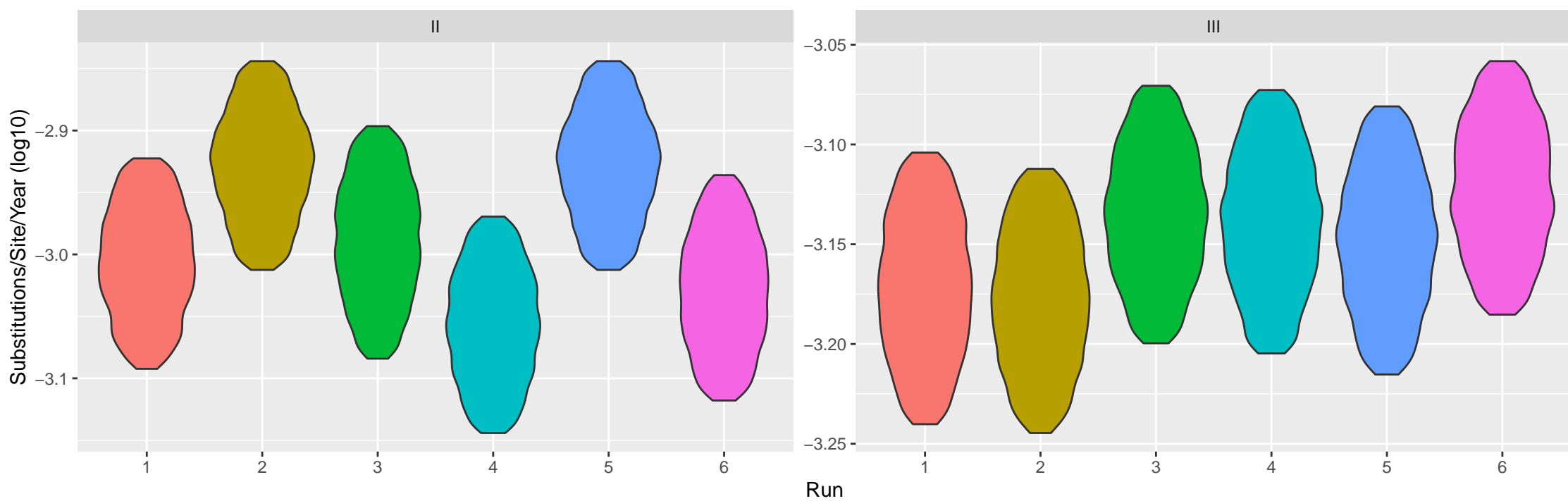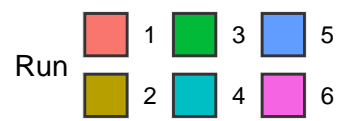

Supplement: Supplementary file 3 [file Data_Sheet_1.zip › Supplementary figure 2.pdf]

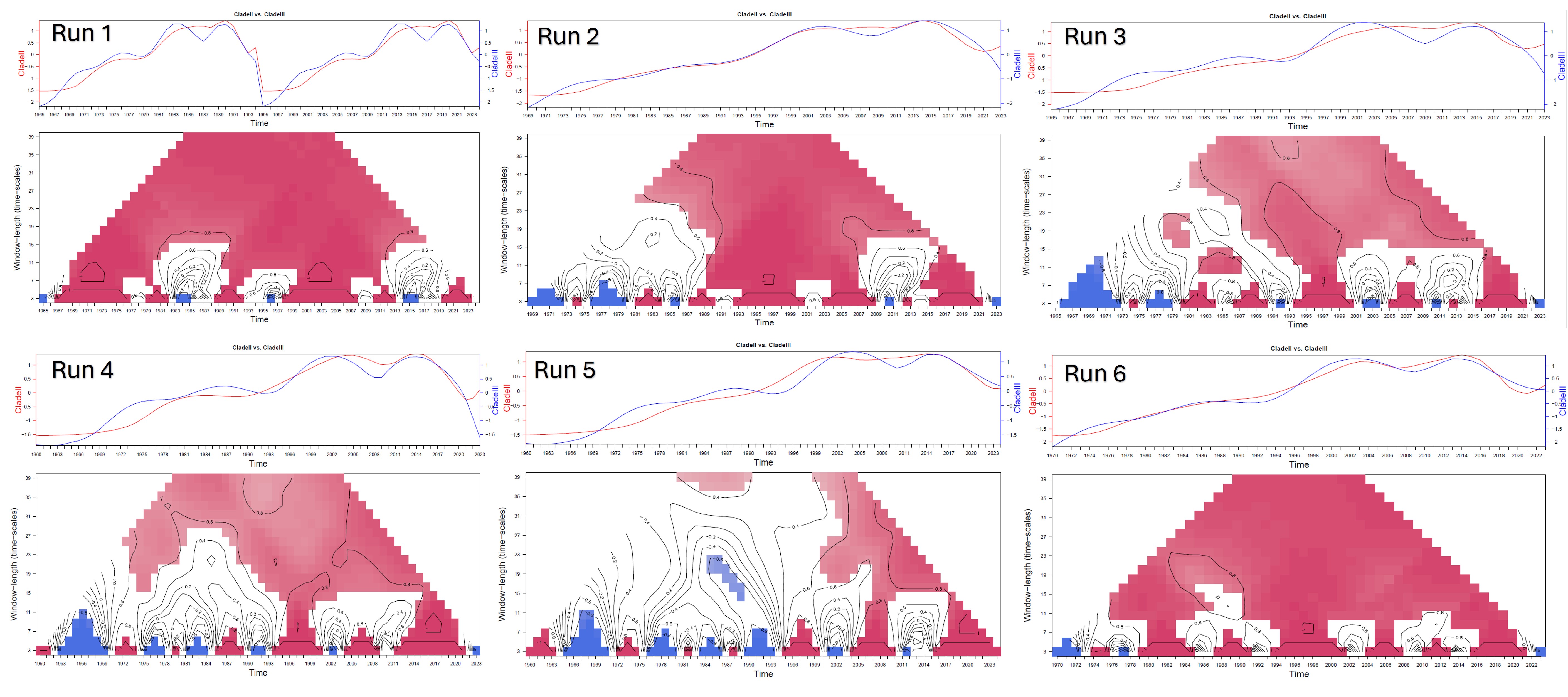

Supplement: Supplementary file 3 [file Data_Sheet_1.zip › Supplementary figure 3.jpg]

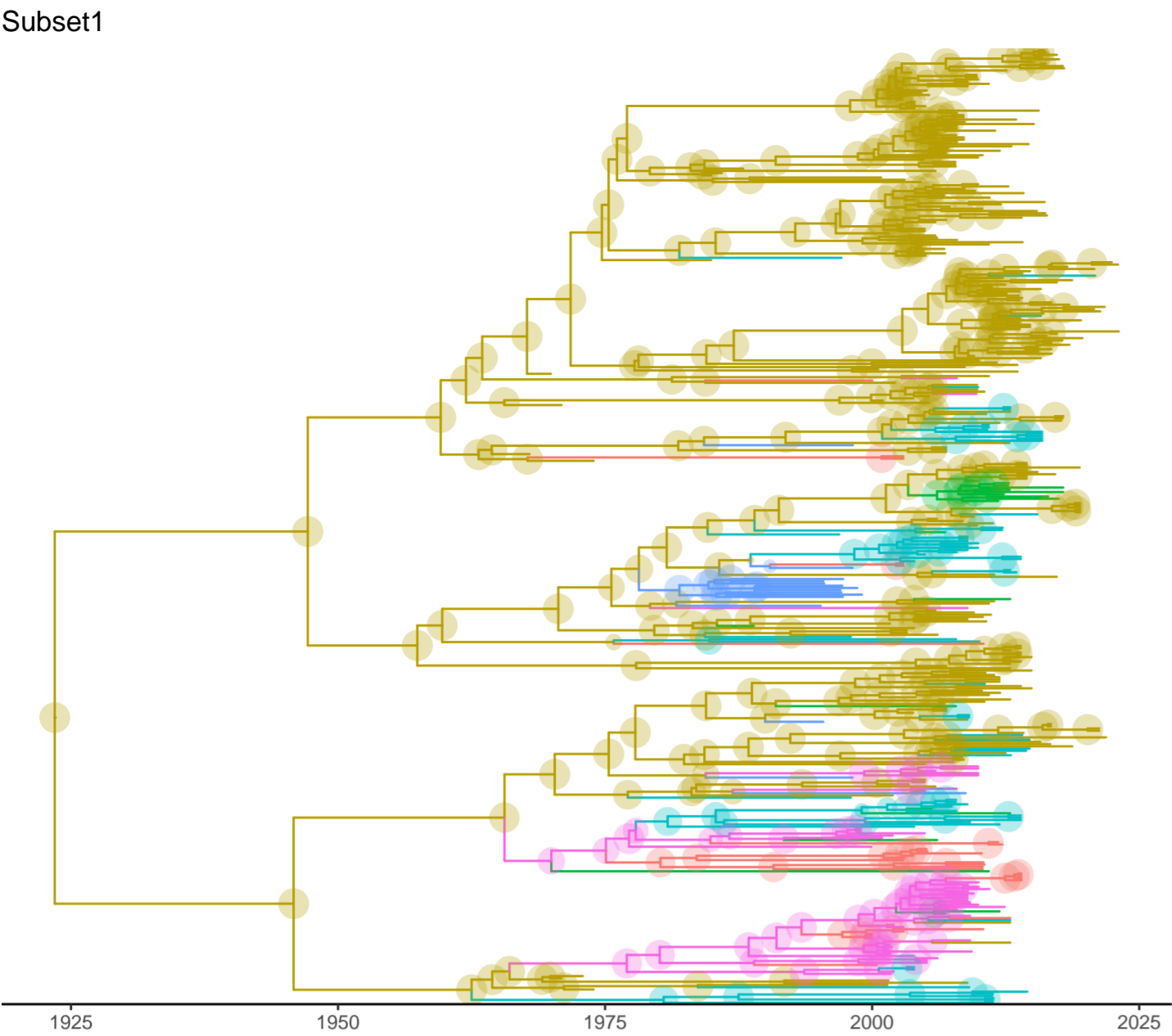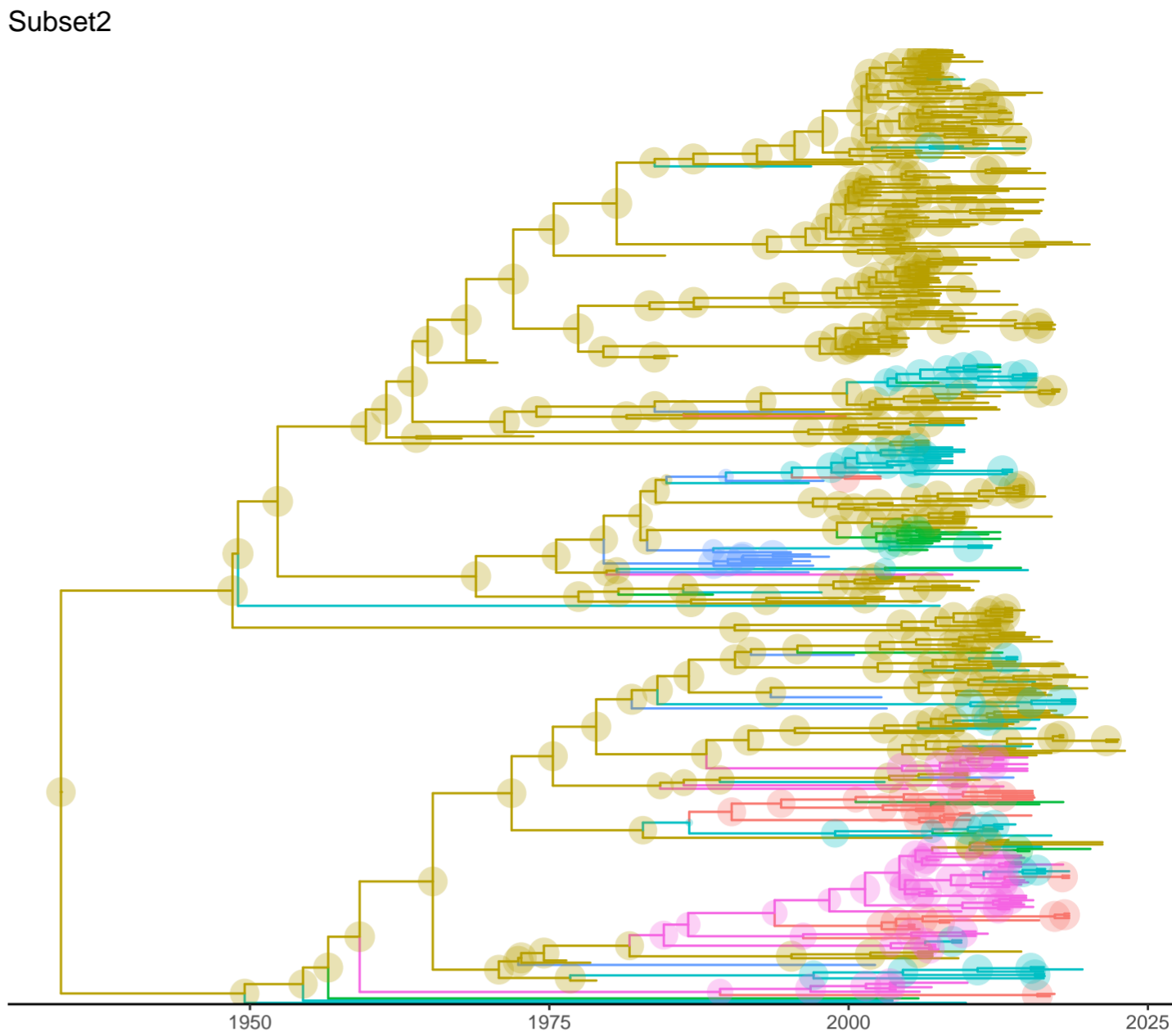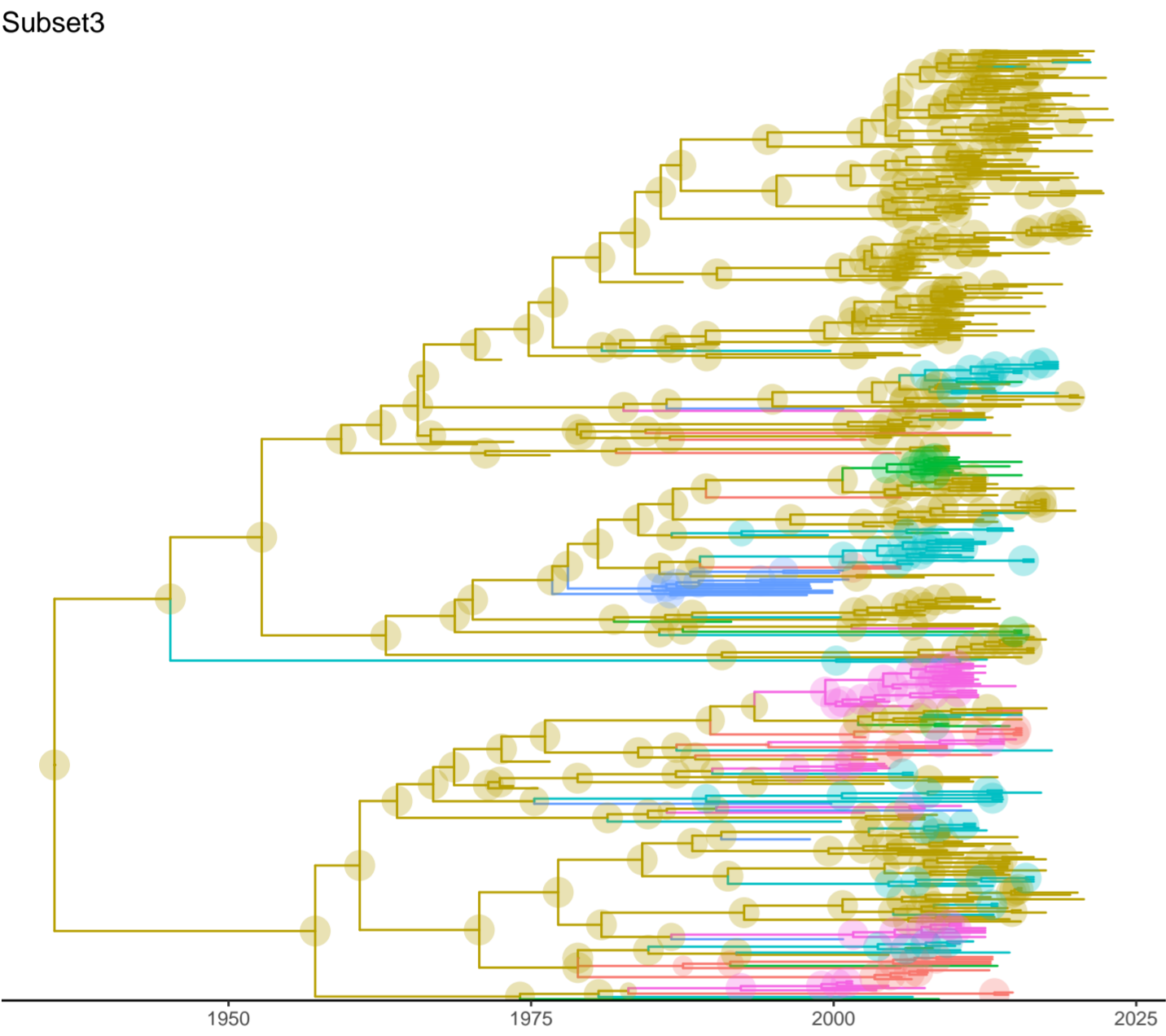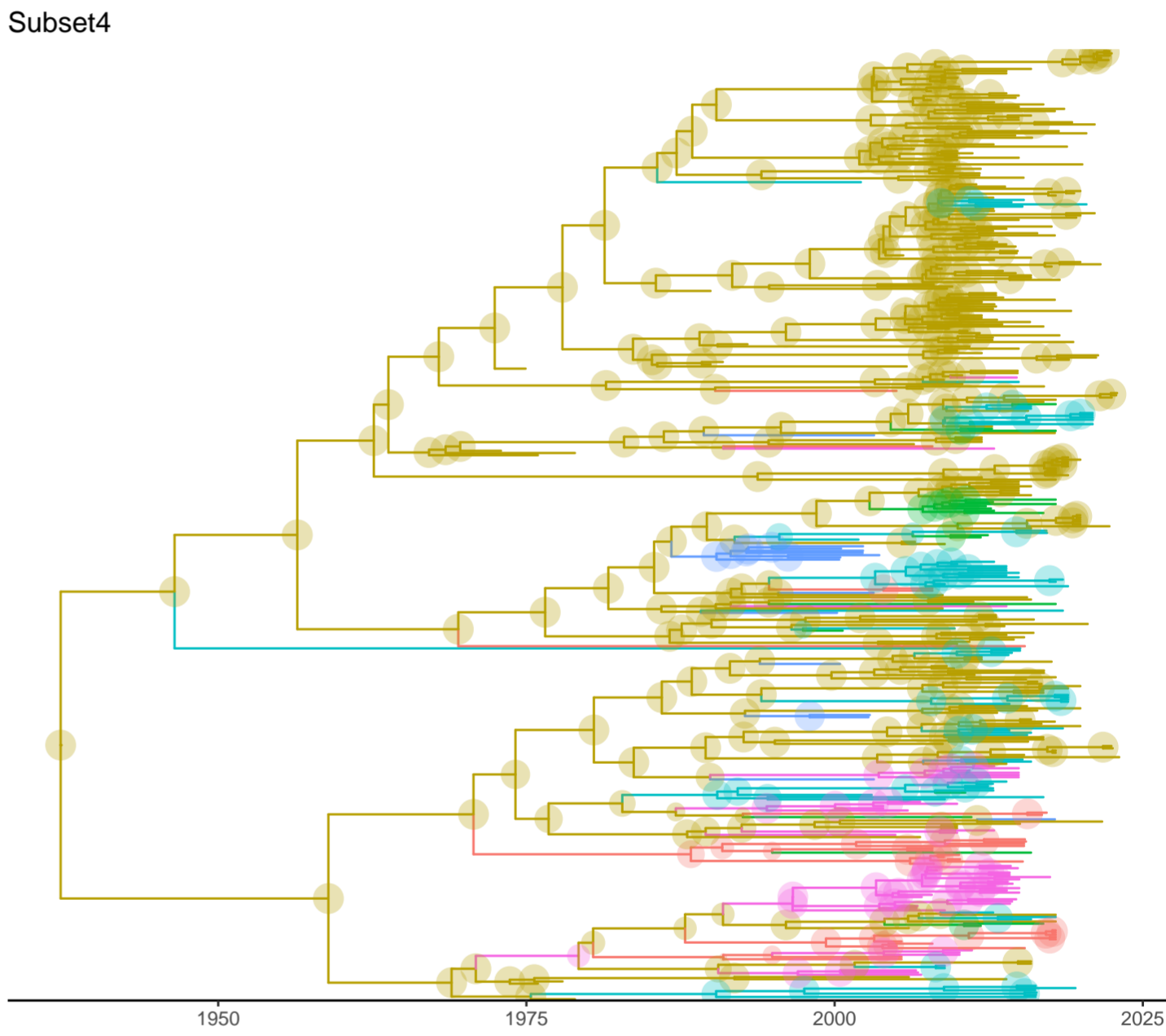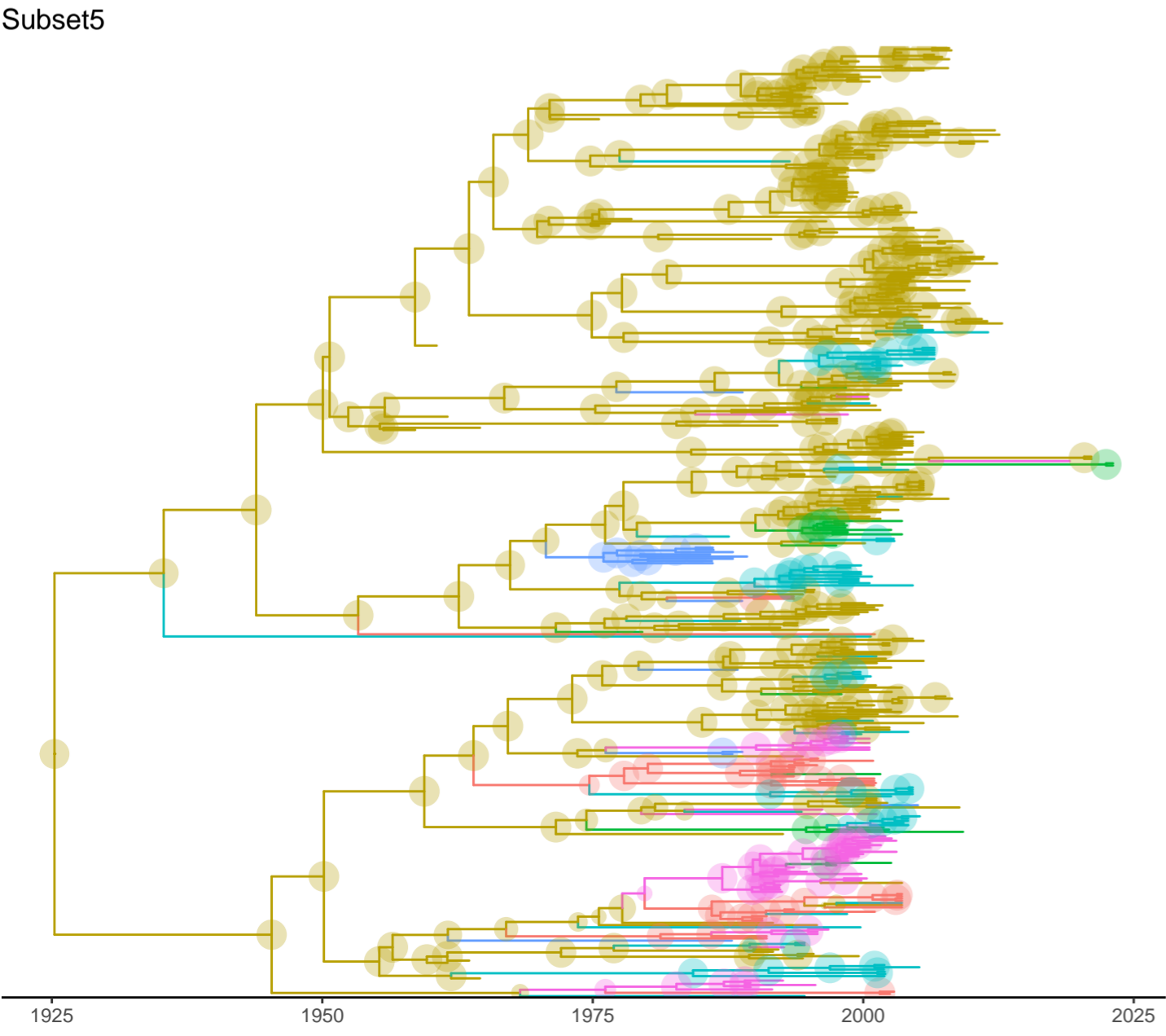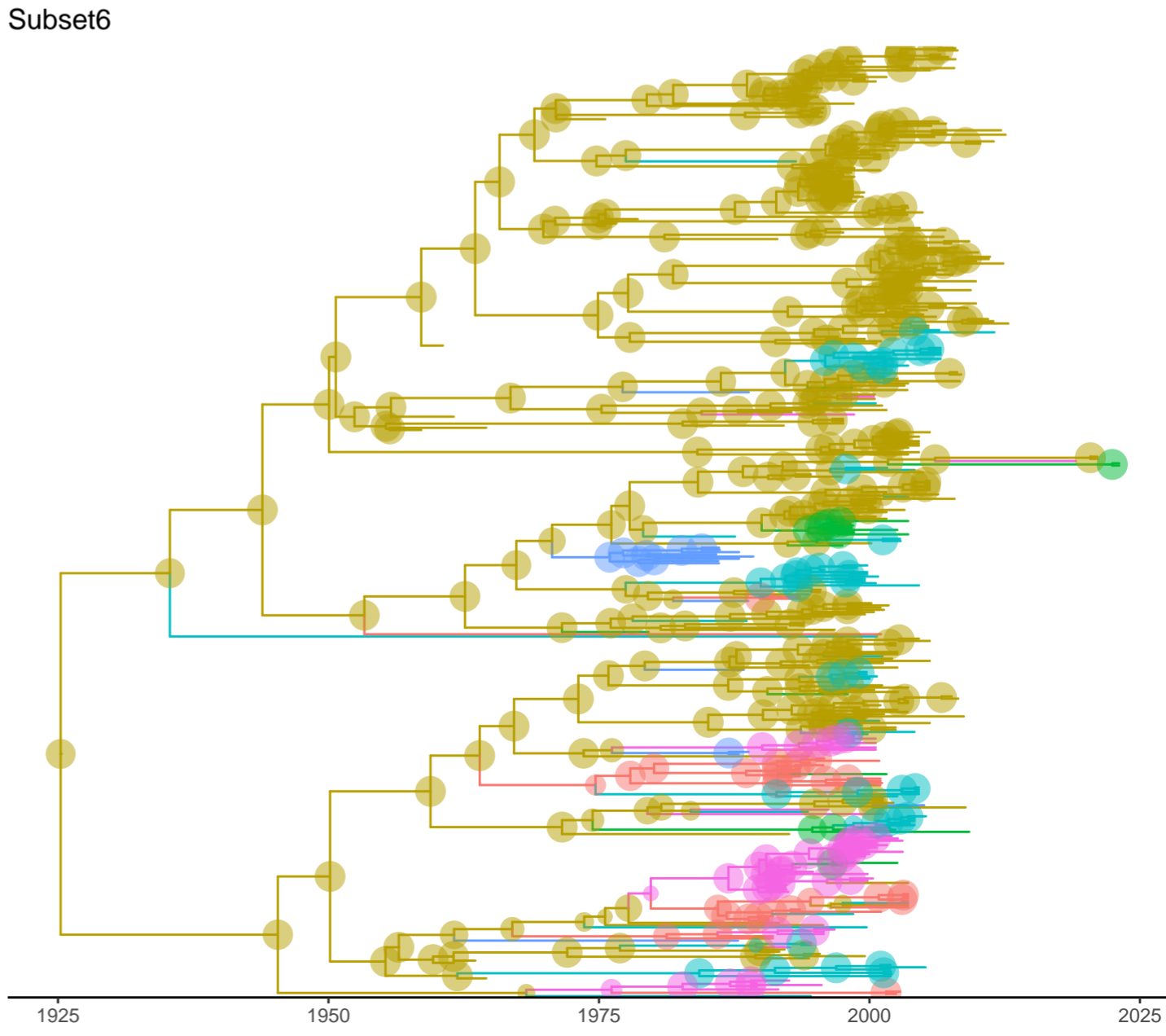

Location

|        |            |              |
|--------|------------|--------------|
| Africa | Europe     | NorthAmerica |
| Asia   | MiddleEast | SouthAmerica |

Supplement: Supplementary file 3 [file Data_Sheet_1.zip › Supplementary figure 4.pdf]

Cladell Run2

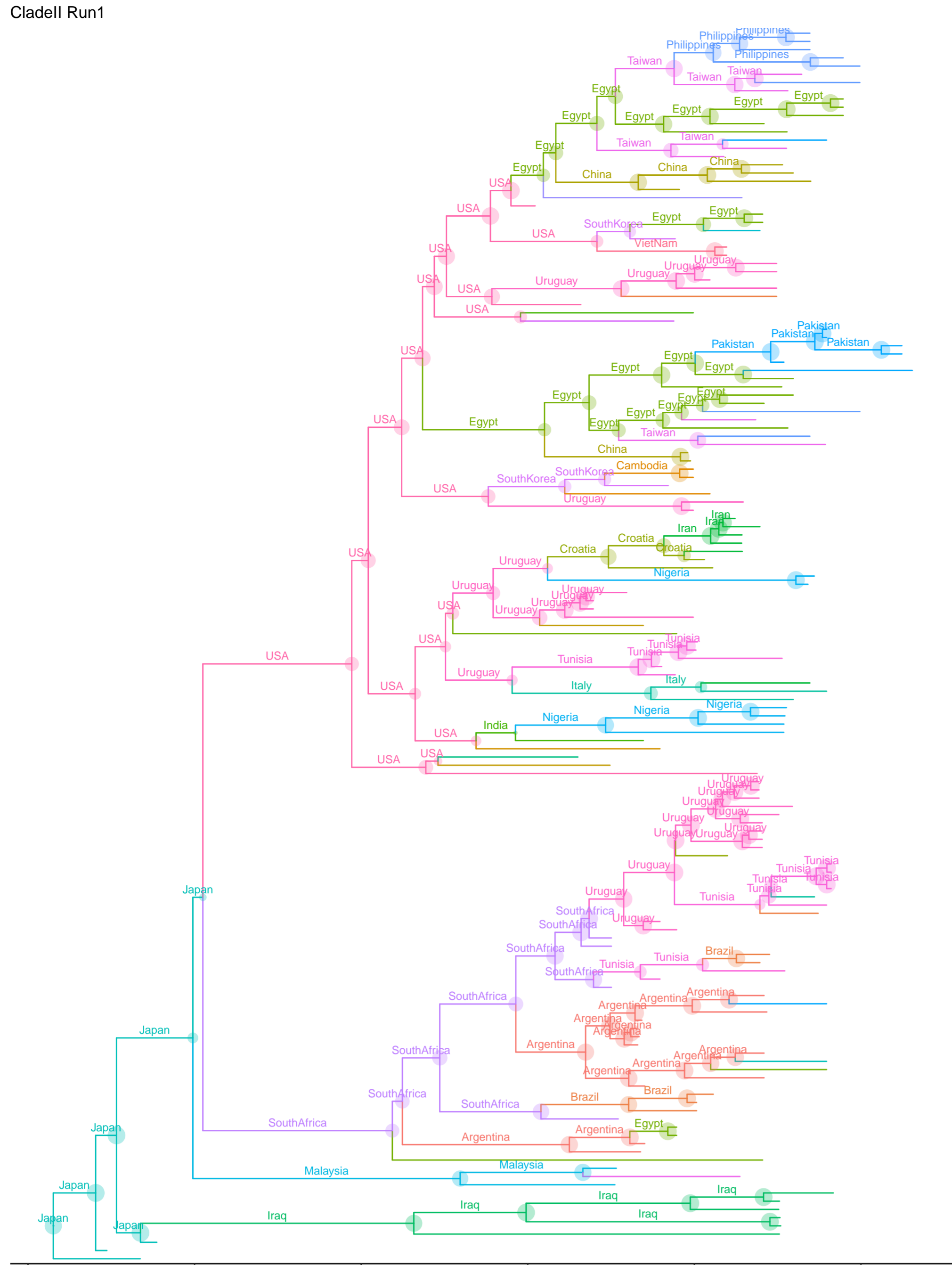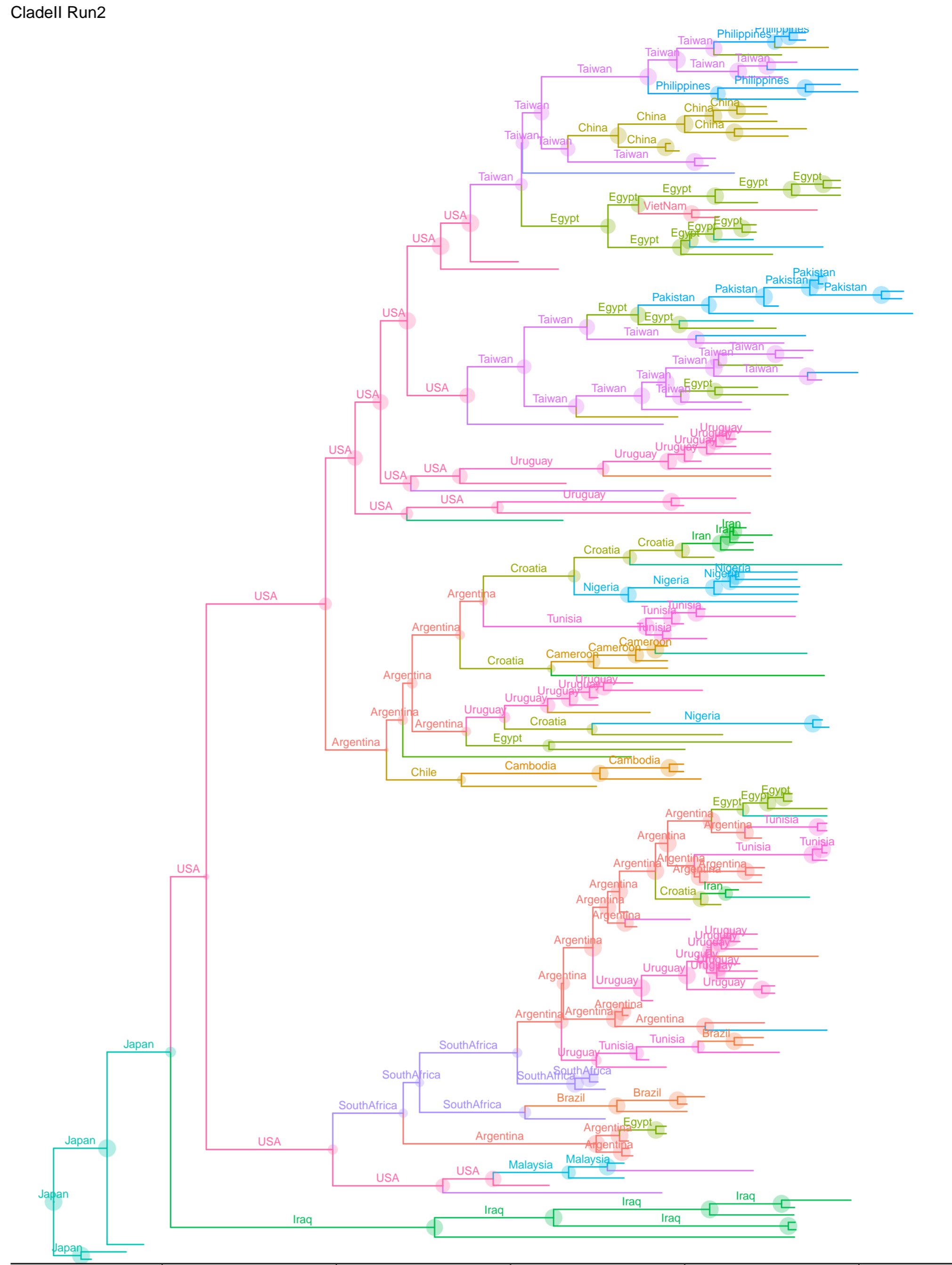

Cladell Run3

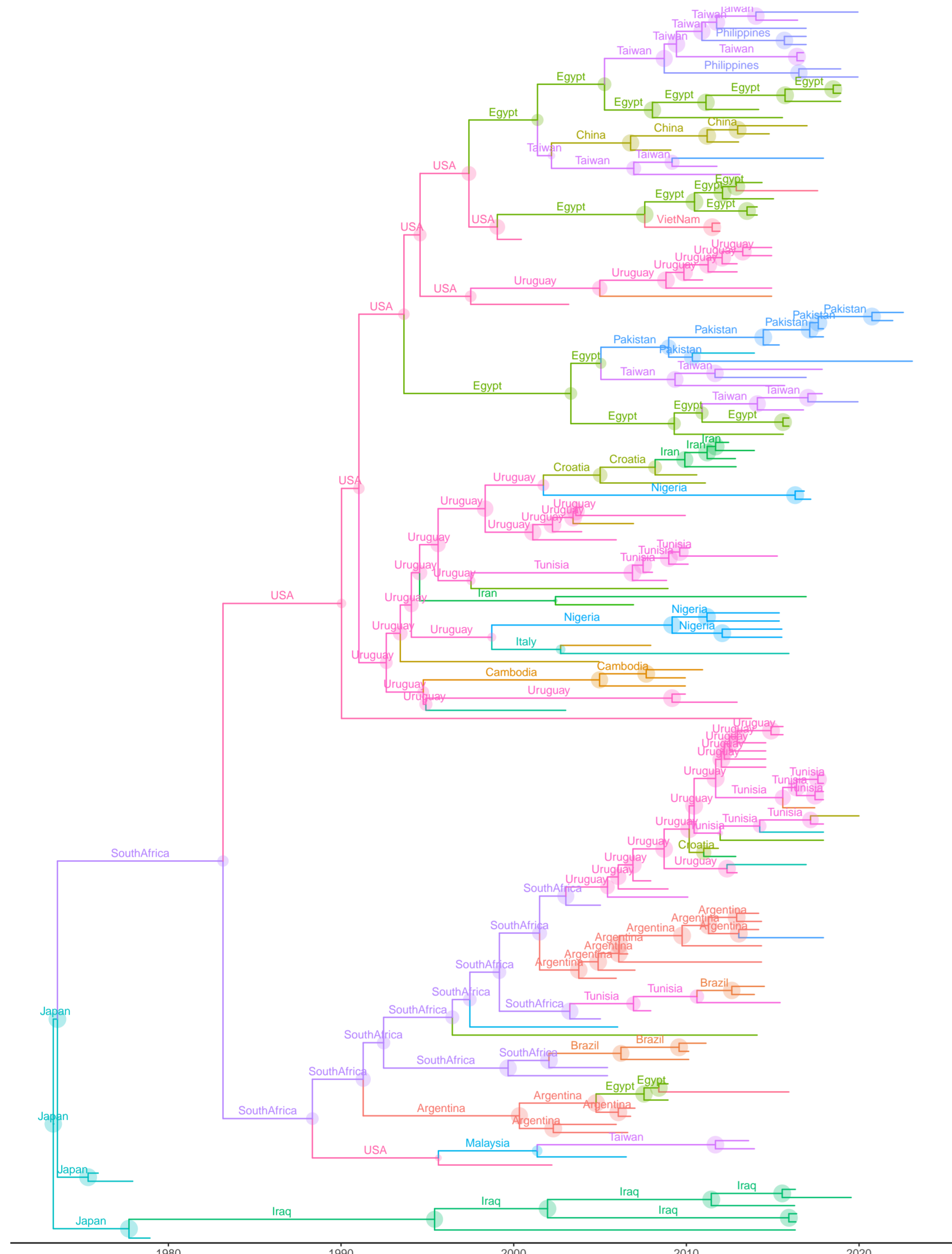

Cladell Run4

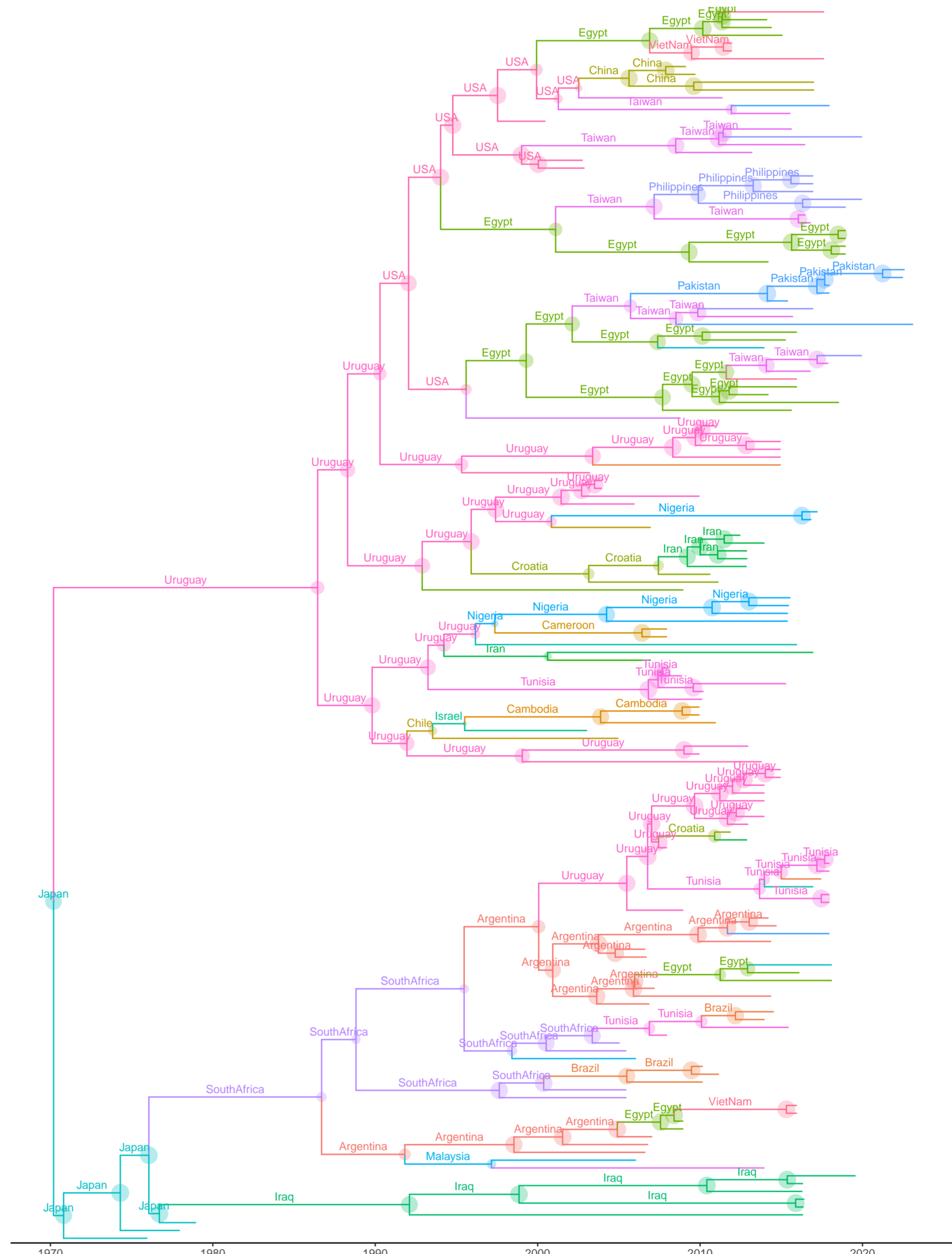

Cladell Run5

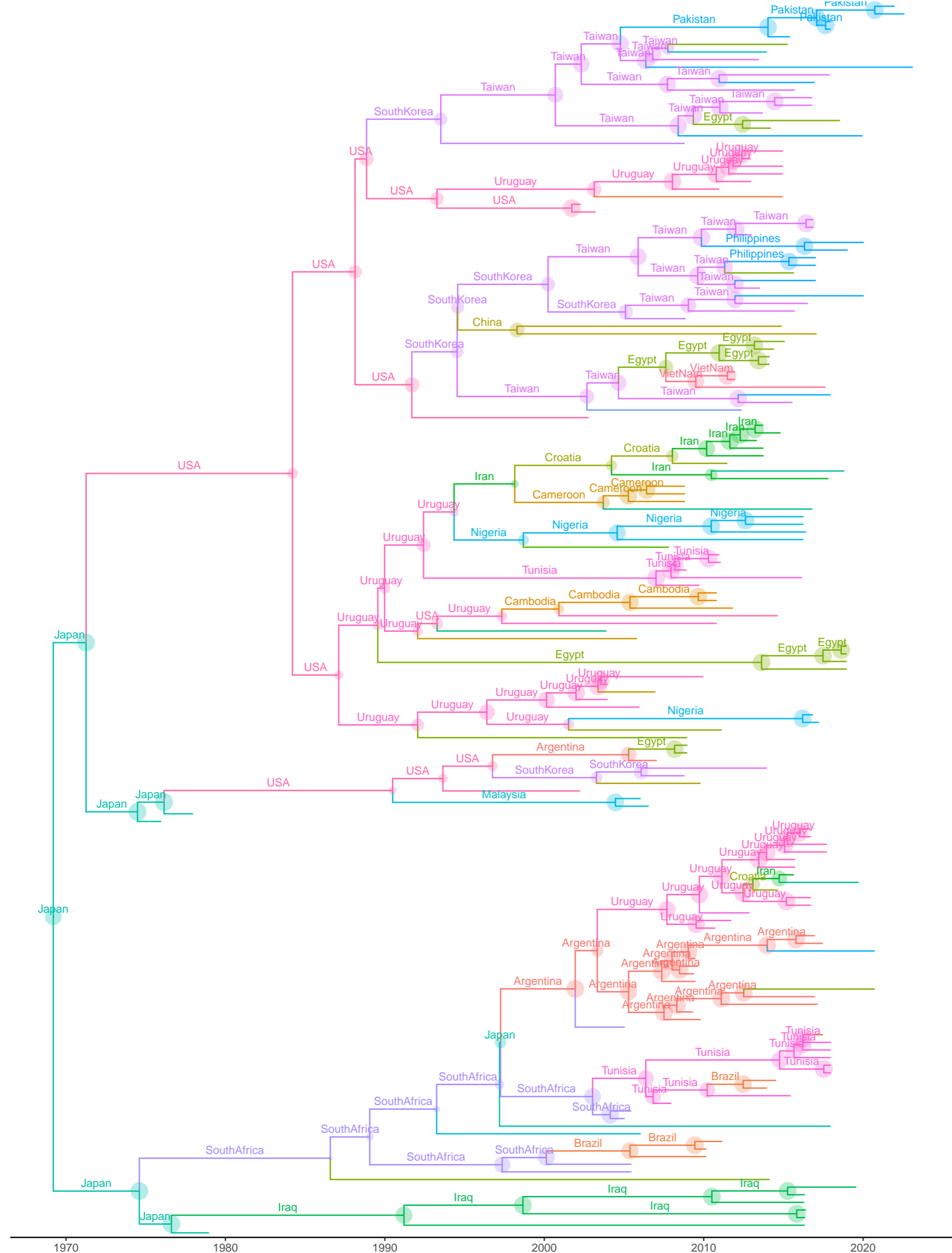

Cladell Run6

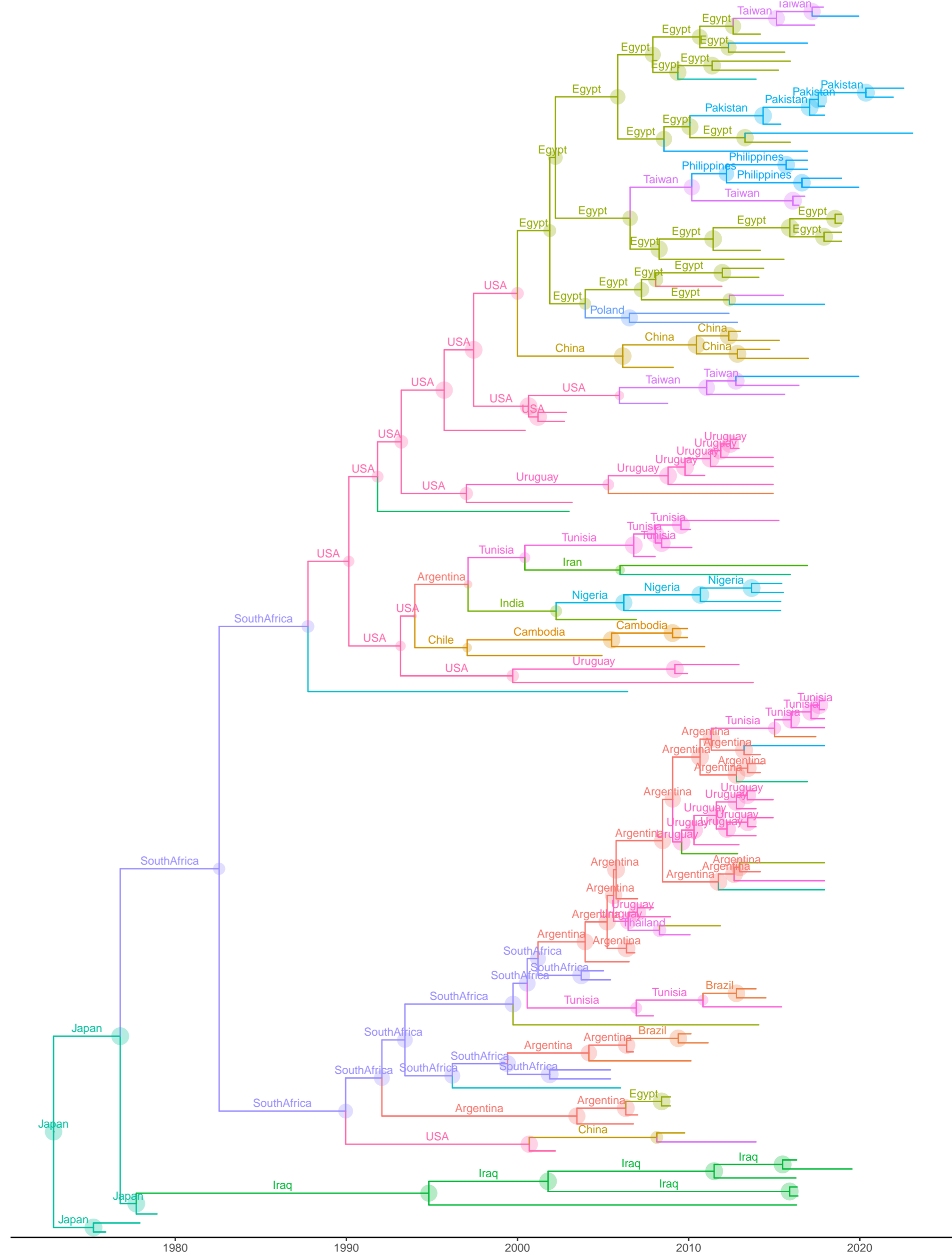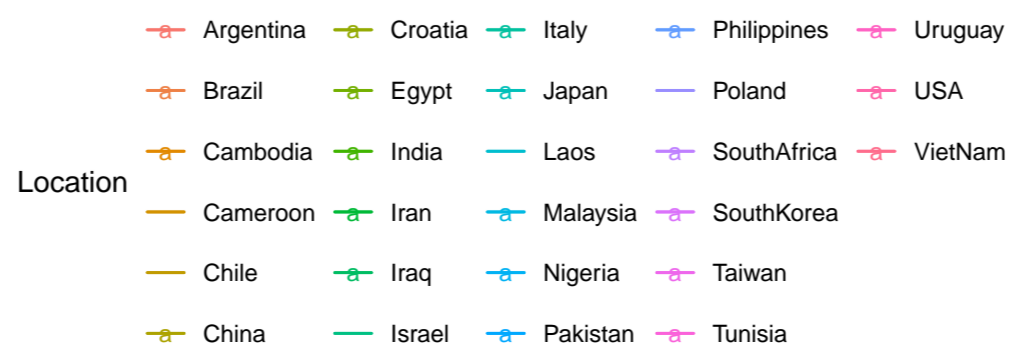

Supplement: Supplementary file 3 [file Data_Sheet_1.zip › Supplementary figure 5.pdf]
